# Exploitation of knowledge databases in the synthesis of zinc(II) malonates with photo-sensitive and photo-insensitive *N*,*N*′-containing linkers

**DOI:** 10.1107/S2052252518001641

**Published:** 2018-03-27

**Authors:** Ekaterina N. Zorina-Tikhonova, Aleksandr S. Chistyakov, Mikhail A. Kiskin, Aleksei A. Sidorov, Pavel V. Dorovatovskii, Yan V. Zubavichus, Eugenia D. Voronova, Ivan A. Godovikov, Alexander A. Korlyukov, Igor L. Eremenko, Anna V. Vologzhanina

**Affiliations:** a N. S. Kurnakov Institute of General and Inorganic Chemistry RAS, Leninskii Pr., 31, Moscow, 119991, Russian Federation; b National Research Center Kurchatov Institute, Ploshchad’ Akademika Kurchatova, 1, Moscow, 123098, Russian Federation; c A. N. Nesmeyanov Institute of Organoelement Compounds RAS, Moscow, 119991, Russian Federation; d Pirogov Russian National Research Medical University, Ostrovityanov Street, 1, Moscow, 117997, Russian Federation

**Keywords:** crystal engineering, crystal structure predictions, coordination polymers, organic solid-state reactions, cycloaddition reactions, single-crystal-to-single-crystal transformations, photo-sensitive ligands

## Abstract

Photoinitiated solid-state reactions are known to affect the physical properties of coordination polymers, such as fluorescence and sorption behaviour, and also afford extraordinary architectures (*e.g.* three-periodic structures with polyorganic ligands). However, the construction of novel photo-sensitive coordination polymers requires an understanding of the factors which govern the mutual disposition of reactive fragments. A series of zinc(II) malonate complexes with 1,2-bis(pyridin-4-yl)ethylene and its photo-insensitive analogues has been synthesized for the purpose of systematic analysis of their underlying nets and mutual disposition of *N*-donor ligands. The application of a big data-set analysis for the prediction of a variety of possible complex compositions, coordination environments and networks for a four-component system has been demonstrated for the first time. Seven of the nine compounds possess one of the highly probable topologies for their underlying nets; in addition, two novel closely related four-coordinated networks were obtained. Complexes containing 1,2-bis(pyridin-4-yl)ethylene and 1,2-bis(pyridin-4-yl)ethane form isoreticular compounds more readily than those with 4,4′-bipyridine and 1,2-bis(pyridin-4-yl)ethylene. The effects of the precursor, either zinc(II) nitrate or zinc(II) acetate, on the composition and dimensionality of the resulting architecture are discussed. For three of the four novel complexes containing 1,2-bis(pyridin-4-yl)ethylene, the single-crystal-to-single-crystal [2 + 2] cycloaddition reactions were carried out. UV irradiation of these crystals afforded either the 0D→1D or the 3D→3D transformations, with and without network changes. One of the two 3D→3D transformations was accompanied by solvent (H_2_O) cleavage.

## Abstract 2

Crystallographic analysis of zinc(II) complexes allows the construction of hydrogen-bonded and coordination networks with 1,2-bis(pyridin-4-yl)ethylene groups situated in photoreactive positions to allow solid-state [2 + 2] cycloaddition reactions.

## 1. Introduction   

**Figure 1:**
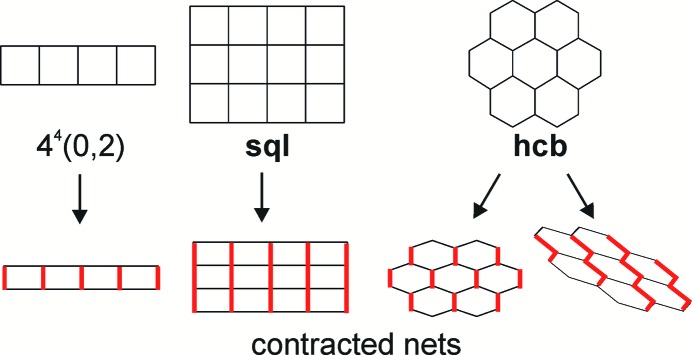
Schematic representation of a ladder [4^4^(0,2)], a square-planar (**sql**) and a honeycomb (**hcb**) net and the shortening of the distance between opposite sides of tetragonal and hexagonal polygons in the case of inequivalent linkers (short edges are red). Herein, the notation of nets is given in accordance with the literature (O’Keeffe *et al.*, 2008 ▸; Blatov *et al.*, 2009 ▸).

Topotactic reactions in the crystals of coordination compounds may proceed keeping (*e.g.* 1D→1D, 2D→2D or 3D→3D) or changing (*e.g.* 0D→1D, 0D→2D, 1D→2D or 2D→3D, including transformations from interpenetrated to non-interpenetrated architectures) the periodicity of the complexes (Vittal, 2007 ▸; Vittal & Quah, 2017 ▸; Huang *et al.*, 2017 ▸). Solid-state reactions involve atomic and molecular movement, which can be accompanied by changes in the properties of a compound such as fluorescence (Papaefstathiou *et al.*, 2004 ▸), luminescence (Hu *et al.*, 2015 ▸), sorption (Sato *et al.*, 2012 ▸) and others. However, the number of types of thermoinitiated topotactic reactions greatly exceeds that of photoinitiated reactions. For example, in the review by Vittal (2007 ▸) only examples of 0D→1D, 1D→1D and 2D→3D transformations are given; since that time, few examples of 1D→3D (Ou *et al.*, 2012 ▸), 1D→2D (Hu *et al.*, 2015 ▸) and 3D→3D (Mir *et al.*, 2010 ▸; Liu *et al.*, 2010 ▸; Michaelides *et al.*, 2011 ▸; Li *et al.*, 2014 ▸) reactions have been published. We consider the insufficient investigation of photoinitiated reactions a result of the factors that govern the mutual disposition of reactive fragments, which are not well understood. With regards to metal complexes, mainly hydrogen bonds (Peedikakkal & Vittal, 2008 ▸) and metallophilic interactions (Georgiev *et al.*, 2010 ▸; Chu *et al.*, 2005 ▸; Schmidbaur & Schier, 2012 ▸) were used to carry out 0D→1D transformations, or in some cases, planar chelate (Papaefstathiou *et al.*, 2004 ▸, 2005 ▸) and bridging (Mir *et al.*, 2010 ▸; Sato *et al.*, 2012 ▸; Nagarathinam & Vittal, 2006 ▸; Chanthapally *et al.*, 2013 ▸; Toh *et al.*, 2005 ▸) carboxylate anions were applied to fix the photo-sensitive species in reactive positions for intramolecular 1D→1D or 2D→2D reactions. For example, 1D coordination polymers with ladder-type structures are characterized by close and parallel orientation of the double bonds in photoreactive spacer ligands (depicted in black in Fig. 1 ▸) if the bridging ligands (shown in red) facilitate the shortening of the metal–metal distances. Solid-state photodimerization in zinc(II) carboxylates containing 1,2-bis(pyridin-4-yl)ethylene (bpe) was carried out (Toh *et al.*, 2005 ▸; Papaefstathiou *et al.*, 2005 ▸); the bridging carboxylate groups make the distance between Zn centers as short as 3.82–3.85 Å. The reduction in distance between opposite linkers is chemically driven and can be carried out for other coordination polymer nets (see the square-planar and honeycomb nets in Fig. 1 ▸).

In our opinion, recent progress in reticular chemistry and data analysis of coordination polymers allows new directions for developing the syntheses of coordination polymers with a desired mutual disposition of photo-sensitive species. The reticular chemistry approach (Yaghi *et al.*, 2003 ▸; Furukawa *et al.*, 2013 ▸) to photoactive materials can be based on the replacement of a photo-insensitive ligand with its photo-sensitive analogue in architectures with desired disposition of reactive fragments. In this case, a chemist should determine which ligands form an isoreticular series. An expert system based on the analysis of all X-rayed compounds allows prediction of the most abundant compositions and architectures of coordination polymers, even for insufficiently explored mixtures (Alexandrov *et al.*, 2011 ▸, 2015*a* ▸,*b* ▸; Mitina & Blatov, 2013 ▸; Schoedel & Zaworotko, 2014 ▸), but cannot predict which of all the possible architectures will form and how 1D and 2D polymers will be situated with respect to each other. Nevertheless, despite various coordination numbers, coordination environments and complex compositions, similar networks can be obtained; also 2D nets such as honeycomb or square-planar (Fig. 1 ▸) and 3D nets such as diamond or primitive-cubic appear much more often than nets with the same connectivity of nodes, making the synthesis of architectures with the desired disposition of ligands possible.

Herein, we discuss the results of our investigation, which aimed to reveal the effectiveness of the reticular chemistry approach and expert system predictions for the construction of zinc(II) coordination polymers with photo-sensitive bpe linkers situated in reactive positions. Zinc(II) aqueous solutions were used because zinc can have various coordination numbers. Photo-insensitive 4,4′-bipyridine (bipy) and 1,2-bis(pyridin-4-yl)ethane (bpa) were used as analogues of bpe to reveal if these ligands could form isoreticular series. We assumed that not only bridging monocarboxylates, but also short bridging dicarboxylate anions are able to distort networks and shorten the distance between the nitrogen-containing ligands (Fig. 1 ▸). Thus, dimethylmalonate (Me_2_mal) and diethylmalonate (Et_2_mal) anions (*An*) were used, as these fix the Zn:*An* ratio to 1:1 (to decrease the number of symmetrically independent anions in the unit cell) and have less coordination modes than the malonate anion (Gogoleva *et al.*, 2017 ▸).

## 2. Results and discussion   

### 2.1. Knowledge-based prediction of complex composition, periodicity and topology in Zn^II^:*L*:*An* ^2−^:H_2_O mixtures   

**Figure 2:**
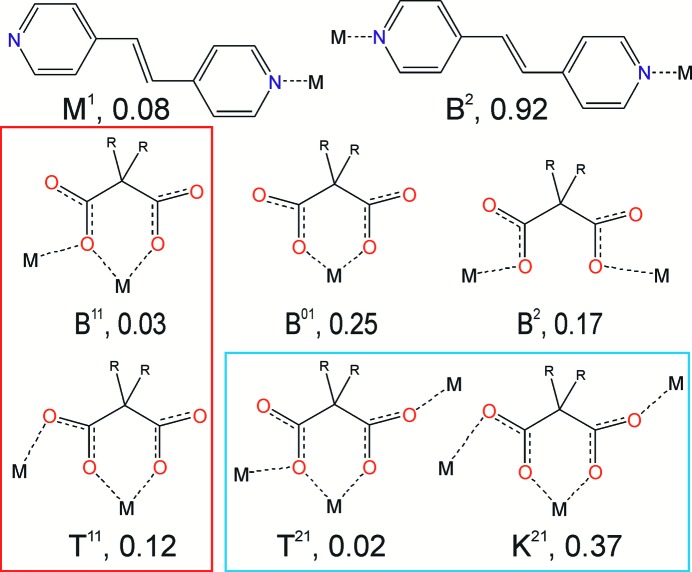
Coordination modes and their associated probabilities of occurrence for bpe or malonate anion and its derivatives in zinc(II) complexes.

An algorithm for the estimation of the most abundant nets of coordination polymers has been described in recent papers (Mitina & Blatov, 2013 ▸; Alexandrov *et al.*, 2015*a* ▸,*b* ▸; Shevchenko *et al.*, 2017 ▸). It is based on the analysis of crystallographic databases to reveal possible coordination numbers and coordination polyhedra of a given metal atom, ratio of cations, anions and neutral ligands which depend on the coordination number, and possible coordination modes of all ligands. Moreover, the probability of the occurrence of a particular motif can be estimated if the number of X-rayed representatives in a mixture is large enough. Let us take Zn^II^:*L*:*An* ^2−^:H_2_O mixtures as an example (*L* = bipy, bpe or bpa; *An* = Me_2_mal or Et_2_mal). The Cambridge Structural Database (Groom & Allen, 2014 ▸; Groom *et al.*, 2016 ▸) contains data for only 12 compounds constructed of zinc(II), 4,4′-bipyridine or its analogues and malonate derivatives (see Table S1 in the supporting information). Eleven of the compounds form an isoreticular series with **ins** topology and a parallel orientation of the *N*,*N*′-containing linkers and long intermolecular distances between their centers (≃ 7.5 Å). Although this net seems to be inappropriate for the construction of photo-sensitive coordination polymers, we expected that the other architectures with close disposition of neutral linkers can appear in this system. In order to reveal all possible motifs in this system and estimate the probability of their occurrence, we examined the data for the crystal structures of similar compounds. Distribution of zinc(II) coordination numbers and the compositions of coordination polyhedra have been obtained from 1473 complexes containing both zinc(II) and a bipy, bpe or bpa ligand. Among them, 220 compounds containing 298 symmetrically independent bpe ligands (the target photo-sensitive ligand) were used to determine the possible coordination modes of this linker and the probable Zn:*L* ratios. Finally, 54 zinc(II) malonates were analyzed to estimate the distribution of coordination modes of the anion. Both bpe and the *An* group are able to crystallize as non-coordinated species or terminal ligands, but this is a rare case more typical for acidic solutions. The other coordination modes found are depicted in Fig. 2 ▸ (cases of terminal anion ligands have not been depicted).

Coordination modes are denoted in accordance with the nomenclature described by Serezhkin *et al.* (2009 ▸). Each ligand *L* is designated by one of the letters *M*, *B*, *T* or *K* depending on the number of donor atoms connected to the metal (1, 2, 3 or 4, respectively). The total number of metal atoms coordinated by the ligand (ν) is given as upper index in the form of the string *mbtk* where each integer *m*, *b*, *t*, *k* is equal to the number of metal atoms coordinated by one, two, three or four donor atoms; ν = *m* + *b* + *t* + *k*. The coordination mode of the ligand is represented by the symbol *L* ^*mbtk*^, and the local topology of the complex group is written as coordination formula (CF) *A*_*n*_ *L*1_*m*_ *L*2_*k*_… where *A* denotes a metal atom, for example, zinc(II), *n* is the number of metal atoms in a polynuclear unit, and *L*1, *L*2… designate the coordination modes of ligands. This compact nomenclature allows us to easily distinguish different types of bridge or chelate ligands, in order to calculate the coordination number of a metal atom and the composition of its coordination polyhedron (see supporting information for details). Based on the coordination formula, further possible structural motifs can be proposed using reported correlations between coordination formulas of complexes and the topologies of coordination polymers (Mitina & Blatov, 2013 ▸; Alexandrov *et al.*, 2015*a* ▸,*b* ▸; Shevchenko *et al.*, 2017 ▸).

**Figure 3:**
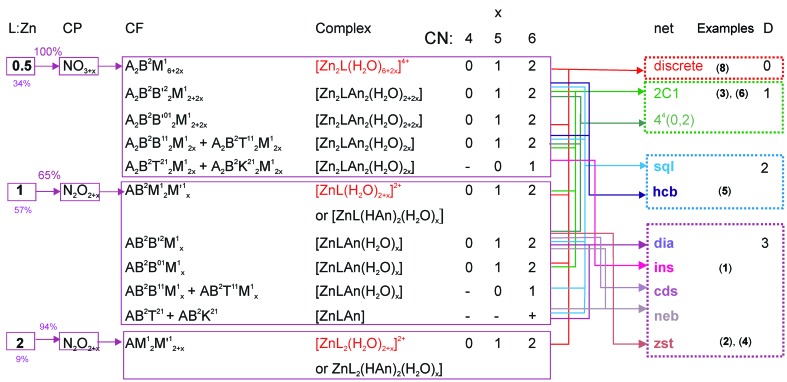
The family of the most probable complexes and their coordination formulas in the Zn^II^:*L*:*An* ^2−^ (or H*An* ^−^):H_2_O mixtures as well as the most probable nets (depicted in Figs. 1 and 4) found for these coordination formulas. CP: coordination polyhedron; CF: coordination formula; CN: coordination number; D: dimensionality of a network; Examples: synthesized compounds which have the topology of the underlying net are given at the left column.

Further analysis of complexes obtained from Zn^II^:*L*:*An* ^2−^:H_2_O mixtures will be limited to those containing: (i) at least one nitrogen atom coordinated to a zinc(II) atom; (ii) a zinc(II) atom with a coordination number of 4, 5 or 6; (iii) a Zn^II^:*L* ratio equal to 2:1, 1:1 or 1:2; (iv) Zn^II^:*An* ^2−^ = 1:1 or Zn^II^:H*An* ^−^ = 1:2. Additional requirements include: (v) if the number of independent zinc atoms or ligands (*A*, *An* or *L*) is equal to 2, both species should have the same coordination mode; (vi) water molecules act as terminal (*M* ^1^) ligands only. The first requirement (i) is needed to control the disposition of nitrogen-containing ligands. The restrictions (ii) and (iii) are true for 98% of all Zn^II^:*L* complexes and 86% of Zn^II^:bpe or bpa complexes, respectively. From requirement (v), it follows that zinc(II) in [Zn_2_ *L*(H*An*)_4_(H_2_O)_*x*_] and [Zn_2_ *L* *An*_2_(H_2_O)_*x*_] complexes have an even number of nitrogen atoms and [Zn*L*_2_ *An*(H_2_O)_*x*_] complexes have an odd number of nitrogen atoms in the coordination polyhedron. The full table of the complexes which satisfy the above criteria, including their coordination formulas and possible nets is given in the supplementary information (Table S2). In Fig. 3 ▸, predictions of the most probable complexes are derived from the following facts: 92% of bpe molecules in zinc complexes act as bridging (*B* ^2^) ligands; H*An* ^−^ tends to act as a terminal ligand or counterion in contrast with *An* ^2−^, which have bridge (*B* ^2^), chelate (*B* ^01^) or bridge-chelate (*B* ^11^, *T* ^11^, *T* ^21^, *K* ^21^) coordination modes mainly (Fig. 2 ▸). Note, that anions in the *B* ^2^, *B* ^11^ and *T* ^11^ or *T* ^21^ and *K* ^21^ coordination modes bind two or three metal atoms in a similar disposition; thus, they form similar complexes, and often similar networks. Fig. 3 ▸ contains the most expected architectures for these complexes, either discrete complexes (for example, dimers of the [Zn_2_ *L*(H_2_O)_6+2*x*_]^4+^ composition), or coordination polymers (one-, two- or three-periodic). Note that polymers can have various *L*:Zn ratios and CFs, but still produce the same topology of the underlying net. For example, both [Zn_2_ *L* *An*_2_(H_2_O)_2+2*x*_] and [Zn*L* *An*(H_2_O)_*x*_] complexes with CFs, *A*_2_ *B* ^2^ *B* ^2^_2_ *M* ^1^_2+2*x*_ and *AB* ^2^ *B* ^2^ *M* ^1^_*x*_ (*x* = 0, 1 or 2), respectively, can belong to the set of the one-periodic ladder 4^4^(0,2) or two-periodic square-planar **sql** architectures.

As shown by Fig. 3 ▸, discrete complexes or complexes with simple chain structures are mainly found in acidic solutions (corresponding chemical formulas are marked in red). Similar architectures are typical for complexes with chelate (*B* ^01^) anions, as these do not act as linkers. The complexes containing bridge (*B* ^2^) anions and 4,4′-bipyridine analogues are able to give one-, two- and three-periodic coordination polymers, including the widespread chain (2*C*-1), ladder [4^4^(0,2)], **sql**, **hcb**, **dia** and **cds** nets [see Mitina & Blatov (2013 ▸) and Alexandrov *et al.* (2011 ▸) for the analysis of the distribution of two- and three-periodic nets]. A characteristic feature of these nets and the **neb** and **zst** networks is that all ligands including the anions act as linkers. Nets such as **ins** require an anion to connect three zinc atoms in order to have the *T* ^21^ or *K* ^21^ coordination mode. As illustrated in Figs. 1 ▸ and 4 ▸, nets including ladder 4^4^(0,2), **sql**, **hcb**, **ins**, **cds** and **zst** allow realisation of close and parallel disposition between linkers if the net is distorted by some short linkers. For **dia** and **neb** nets, close disposition within the net is hardly possible, although it can appear between parallel linkers from interpenetrated nets. To summarise, there is a high probability of two- and three-periodic ladder architectures with parallel orientation of ethenyl groups in Zn^II^:*R*_2_mal^2−^:bpe:H_2_O mixtures, because the malonate anion and its alkyl derivatives favour close disposition of zinc(II) cations and all ligands connected with them.

The validity of above approach is verified by the fact that a number of previously obtained zinc(II) dicarboxylates containing 4,4′-bipyridine and its analogues are characterized by the above CFs and have one of the proposed topologies (Table S2). Moreover, a number of zinc(II) nitrate (Peedikakkal & Vittal, 2008 ▸), monocarboxylate (Toh *et al.*, 2005 ▸; Chanthapally *et al.*, 2014 ▸) or dicarboxylate (Mir *et al.*, 2010 ▸; Sato *et al.*, 2012 ▸; Ou *et al.*, 2011 ▸, 2012 ▸; Hu *et al.*, 2015 ▸) complexes with bpe have the above topologies and are known to undergo solid-state photochemical [2 + 2] cycloadditions. Particularly, dicarboxylates with the compositions: [Zn(bpe)(muco)]·DMF·H_2_O and [Zn(bpe)(1,4-bdc)]·DMF (Mir *et al.*, 2010 ▸), [Zn_2_(bpe)(1,2-bdc)_2_] (Ou *et al.*, 2011 ▸), [Zn(bpe)(moip)]·DMF (Sato *et al.*, 2012 ▸), [Zn(bpe)_2_(1,2-chdc)(H_2_O)_2_] (Ou *et al.*, 2012 ▸) and [Zn(bpe)(*L*)(H_2_O)] (Hu *et al.*, 2015 ▸) are characterized by their CF and net, respectively, *AB* ^2^ *K* ^21^/**fet**, *A*_2_ *B* ^2^ *B* ^11^_2_/4^4^(0,2), *AB* ^2^ *K* ^21^/3,5*L*2, *AM* ^1^_2_ *B*_2_ *M*′^1^_2_/2*C*-1 and *AM* ^1^ *B* ^2^ *M*′^1^/2*C*-1 {H_2_muco = *trans*,*trans*-muconic acid, H_2_bdc = benzene dicarboxylic acid; H_2_moip = 5-methoxy­isophthalatic acid; H_2_chdc = cyclohexanedicarboxylate; H_2_ *L* = 4,4′-[1,2-phenylenebis(methylene)bis(oxy)]dibenzoic acid}.

### 2.2. Synthesis   

**Figure 5:**
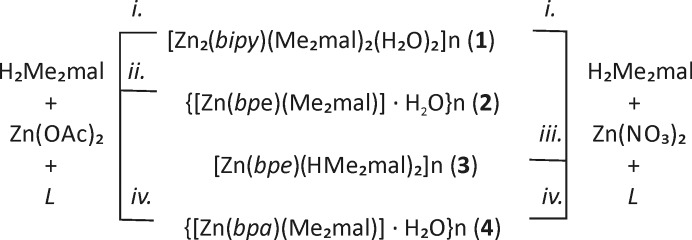
Synthesis of zinc(II) dimethylmalonates (**1**)–(**4**). Conditions: (i) H_2_Me_2_mal (1 eq.), Zn(OAc)_2_ or Zn(NO_3_)_2_ (1 eq.), bipy (2 eq.), H_2_O/MeCN, 1 week; (ii) H_2_Me_2_mal (1 eq.), Zn(OAc)_2_ (1 eq.), bpe (2 eq.), H_2_O/MeCN, 1 week; (iii) H_2_Me_2_mal (1 eq.), Zn(NO_3_)_2_ (1 eq.), bpe (2 eq.), H_2_O/MeCN, 1 week; (iv) H_2_Me_2_mal (1 eq.), Zn(OAc)_2_ or Zn(NO_3_)_2_ (1 eq.), bpa (2 eq.), H_2_O/MeCN, 1 week.

**Figure 6:**
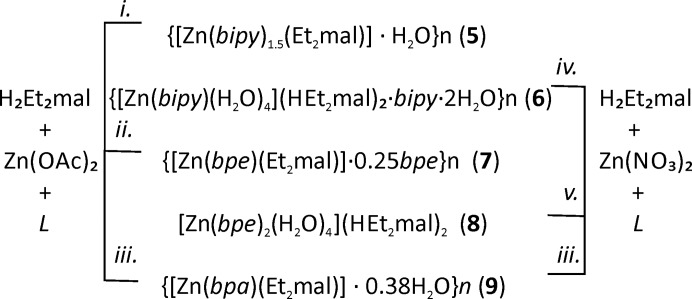
Synthesis of zinc(II) diethylmalonates (**5**)–(**9**). Conditions: (i) H_2_Et_2_mal (1 eq.), Zn(OAc)_2_ (1 eq.), bipy (2 eq.), H_2_O/MeCN, 2 weeks; (ii) H_2_Et_2_mal (1 eq.), Zn(OAc)_2_ (1 eq.), bpe (2 eq.), H_2_O/MeCN, 1 week; (iii) H_2_Et_2_mal (1 eq.), Zn(OAc)_2_ or Zn(NO_3_)_2_ (1 eq.), bpa (2 eq.), H_2_O/MeCN, 1 week; (iv) H_2_Et_2_mal (1 eq.), Zn(NO_3_)_2_ (1 eq.), bipy (2 eq.), H_2_O/MeCN, 1 month; (v) H_2_Et_2_mal (1 eq.), Zn(NO_3_)_2_ (1 eq.), bpe (2 eq.), H_2_O/MeCN, 1 week.

The reaction of zinc(II) nitrate or zinc(II) acetate with dimethylmalonic or diethylmalonic acid in the presence of a nitrogen-containing ligand such as bipy, bpe or bpa afforded nine novel compounds (Figs. 5, 6). Synthesis was carried out by the slow mixing of solutions. Attempts to use a direct synthesis (mixing the reagents in an appropriate solvent) as well as the use of solvothermal methods were unsuccessful, since in both cases we failed to obtain single crystals suitable for X-ray diffraction. The results obtained indicate that the initial zinc(II) salt source can affect the chemical composition of precipitate complexes. All acidic salts and hence, the cationic complexes, were obtained from zinc(II) nitrate. However, starting from zinc(II) acetate, anhydrous neutral complexes more readily formed. Only bpa-containing mixtures (for both Me_2_mal^2−^ and Et_2_mal^2−^) and the Zn^II^:bipy:Me_2_mal^2−^ gave the same products regardless of the cation source.

### 2.3. Crystallography   

Compounds obtained were characterized by means of single-crystal and powder X-ray diffraction techniques. Powder X-ray diffraction confirmed that the samples were phase pure and consisted mainly of the above reaction products (see supporting information). Single-crystal XRD confirmed the validity of our proposal about possible Zn^II^:*L*:*An* ^2−^ ratios and coordination formulas. Only [Zn_2_(bipy)_3_(Et_2_mal)_2_] (**5**) has an unexpected 2:3:2 ratio with two different coordination modes of the bipy ligands, but even in this case the complex can be regarded as an analogue of the [Zn_2_ *L* *An*_2_(H_2_O)_2_] complex with the *A*_2_ *B* ^2^ *B* ^2^_2_ *M* ^1^_2_ CF. Four of the nine complexes obtained [(**2**), (**4**), (**7**), (**9**)] have the ratio Zn^II^:*L*:*An* ^2−^ = 1:1:1 with ligands and anions in bridge mode (and the CF = *AB* ^2^ *B* ^2^). Among these, only [Zn(bpe)(Me_2_mal)]·H_2_O (**2**) and [Zn(bpa)(Me_2_mal)]·H_2_O (**4**) are isostructural (Table S3) and have the same underlying net; however, the networks of (**7**) and (**9**) differ in the disposition of neutral linkers (see below). In [Zn(bpe)(HMe_2_mal)_2_] (**3**) and [Zn(bipy)(H_2_O)_4_](HEt_2_mal)_2_·bipy·2H_2_O (**6**) an anion is protonated, so the Zn^II^:*L*:H*An* ^−^ ratio is equal to 1:1:2, and only a neutral *N*,*N*′-containing ligand acts as a linker, while the anion is a terminal ligand or counterion (the CFs are: *AB* ^2^ *M* ^1^_2_ and *AB* ^2^ *M* ^1^_4_, respectively). In [Zn(bpe)_2_(H_2_O)_4_](HEt_2_mal)_2_ (**8**) the anion is also protonated and acts as a counterion, but the ratio Zn^II^:*L* = 1:2 and the CF = *AM* ^1^_2_ *M*′^1^_4_ to give the discrete complex only. At last, [Zn_2_(bipy)(Me_2_mal)_2_(H_2_O)_2_] (**1**) belongs to the family of complexes with Zn^II^:*L*:*An* ^2−^ = 2:1:2 with the *A*_2_ *B* ^2^ *K* ^21^_2_ *M* ^1^_2_ CF. Note that all these CFs are among the most expected for Zn^II^:*L*:*An* ^2−^:H_2_O mixtures (Fig. 3 ▸).

The discrete [Zn(bpe)_2_(H_2_O)_4_](HEt_2_mal)_2_ (**8**) complex can be regarded as representative of the family of O—H⋯N bonded compounds with parallel and close orientation of the neighbouring bpe or bipy molecules (Table S2). Similar to its nitrate-containing analogue, [Zn(bpe)_2_(H_2_O)_4_](NO_3_)_2_·8/3H_2_O·2/3bpe (Peedikakkal & Vittal, 2008 ▸), it readily undergoes the [2 + 2] cycloaddition 0D→1D photoreaction to give a coordination polymer (see §2.4 for details).

**Figure 7:**
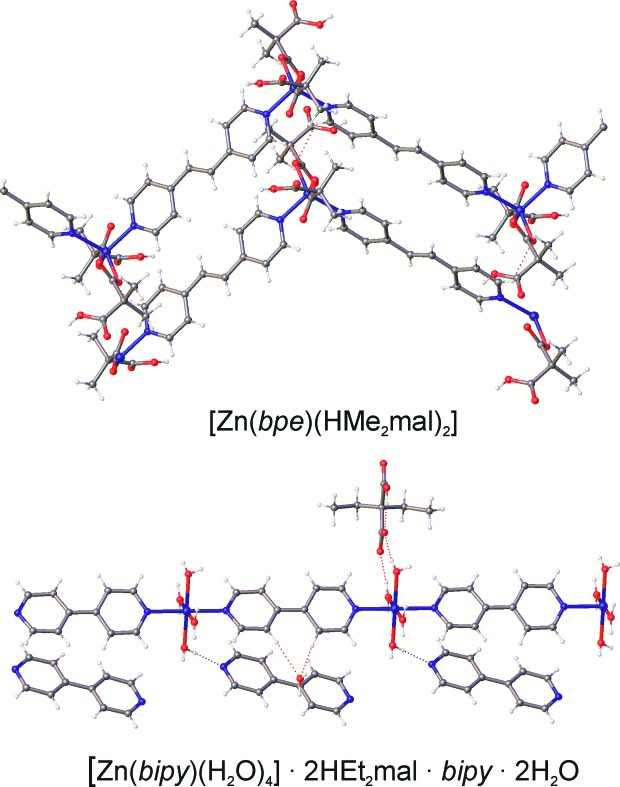
Fragment of [Zn(bpe)(HMe_2_mal)_2_] (**3**) [*r*(Zn–Zn) = 13.5 Å, ∠(Zn–Zn–Zn) = 108°] and [Zn(bipy)(H_2_O)_4_](HEt_2_mal)_2_·bipy·2H_2_O (**6**) [*r*(Zn–Zn) = 11.4 Å, ∠(Zn–Zn–Zn) = 180°] chains.

[Zn(bpe)(HMe_2_mal)_2_] (**3**) and [Zn(bipy)(H_2_O)_4_](HEt_2_mal)_2_·bipy·2H_2_O (**6**) are simple chains with CFs *AB* ^2^ *M* ^1^_2_ and *AB* ^2^ *M* ^1^_4_, respectively (Fig. 7 ▸). As zinc(II) has tetrahedral and octahedral coordination in these complexes, the chains are zigzag and linear, respectively. The chains of [Zn*L*(H_2_O)_4_] composition are always linear with nitrogen atoms of the two linkers situated in the apical position of octahedral zinc(II); the coordinated water molecules are involved in hydrogen bonding with counterions or solvent molecules, which separate *L* from one another. Thus, in such systems photoreactions would be possible if either both *L* and the counterion are UV-sensitive and are situated in positions appropriate for a [2 + 2] cycloaddition reaction, or additional photoreactive *L* molecules are stabilized by O—H⋯N hydrogen bonds in reactive positions with coordinated ones. The head-to-tail packing of zigzag chains of the [Zn*L* *An*_2_] composition is not forbidden in general; there are a few examples with bipy ligands and close parallel disposition of planar ligands [see Mulijanto *et al.* (2017 ▸) for example of photoreactive zigzag chains], but it requires small or linear anions. Bulky anions typically prevent packing of bipy and its analogues in reactive positions, as in the case of (**3**).

**Figure 8:**
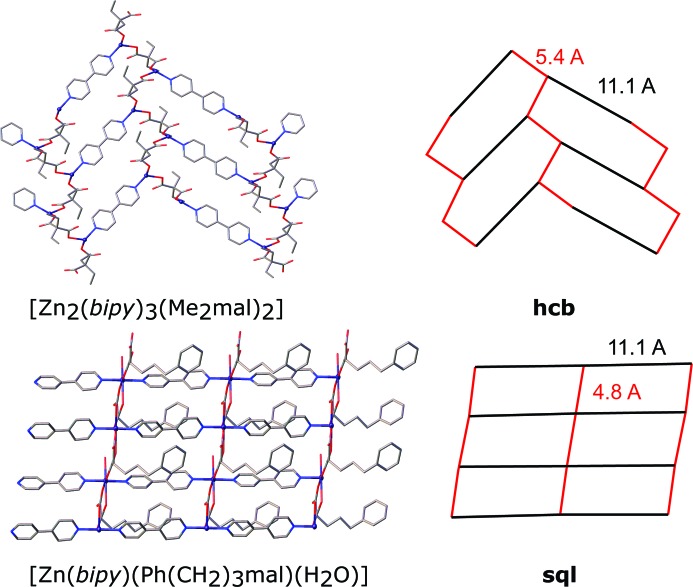
Fragment of the crystal packing in [Zn_2_(bipy)_3_(Me_2_mal)_2_] (**5**) and [Zn(bipy)(Ph(CH_2_)_3_mal)(H_2_O)]·4H_2_O (Gao *et al.*, 2012 ▸) and the underlying nets depicted in black (for bipy linkers) and red (for anions). Hydrogen atoms, terminal ligands and solvent molecules have been omitted.

[Zn_2_(bipy)_3_(Et_2_mal)_2_] (**5**) represents layered complexes and has the **hcb** network topology (Fig. 8 ▸). The layer is almost planar with terminal bipy ligands situated on both sides of the layer. The anions connect the metal atoms in a zigzag chain with *r*(Zn⋯Zn) = 5.4 Å and ∠(Zn–Zn–Zn) = 99°; the chains are further bonded by bipy ligands to the **hcb** net with the distance between metal atoms equal to 11.1 Å. Thus, the net is distorted as four of the six sides of the hexagons are shorter than the other two. This shortening makes the distance between parallel bipy ligands as short as 5.5 Å, but it is still too long to allow the occurrence of a photoreaction for a hypothetical bpe analogue. Note that for the **sql** network found in [Zn(bipy)(Ph(CH_2_)_3_mal)(H_2_O)]·4H_2_O (Gao *et al.*, 2012 ▸) with a bridge-chelate Ph(CH_2_)_3_mal anion, this distance is shorter (4.8 Å). Terminal bipy ligands in (**5**) are directed towards the centres of six-membered cycles of (**5**) sheets, so the layers are packed in a way that also prevents close packing of bridge bipy ligands from neighbouring layers. In the case of less bulky terminal ligands, for example, for tetracoordinated zinc(II) atoms in [Zn_2_(bpa)_3_(tpp)_2_]ClO_4_ (Dias de Souza *et al.*, 2015 ▸) [tpp = 3-(2-thienyl)propanoato] bpa ligands are situated in stacks with closest C–C distances of 4.9 Å, affected by the repulsion from hydrogen atoms of ethane moieties. One can propose fewer restrictions in the case of planar bpe ligands and the possibility of [2 + 2] cycloaddition reactions for bpe analogues.

**Figure 9:**
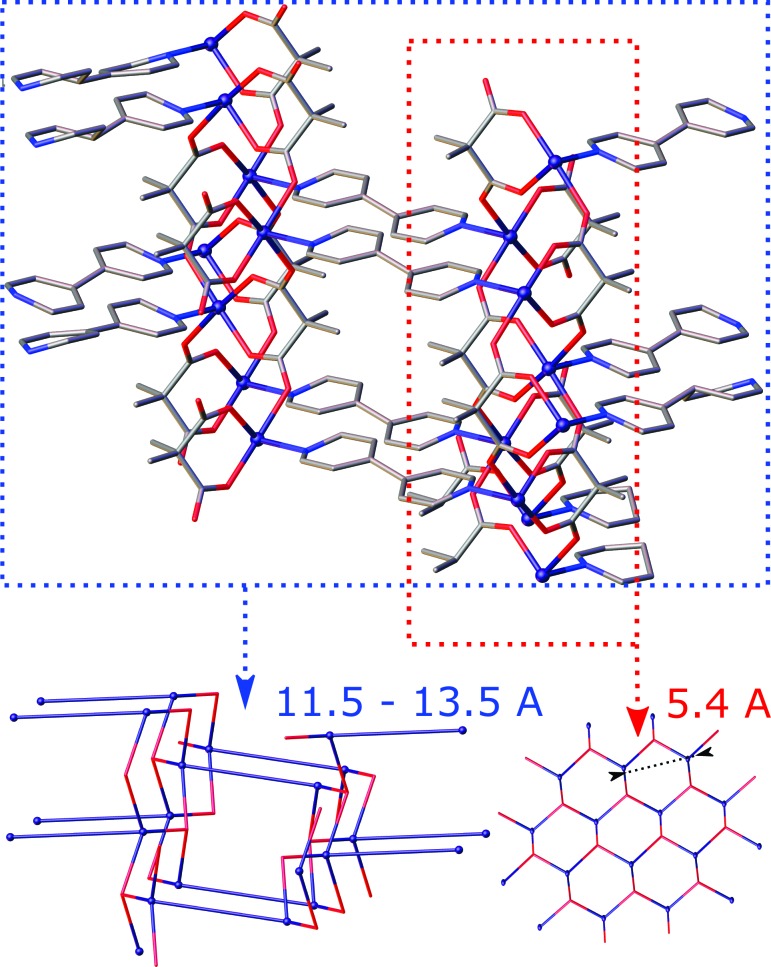
Fragment of the crystal packing in [Zn_2_(bipy)(Me_2_mal)_2_(H_2_O)_2_] (**1**) (top) and the underlying net (bottom) depicted in blue (for bipy linkers) and red (for anions). Hydrogen atoms have been omitted.

The other coordination polymers synthesized are 3D networks. Complex [Zn_2_(bipy)(Me_2_mal)_2_(H_2_O)_2_] (**1**) belongs to the family of zinc(II) malonates with the *A*_2_ *B* ^2^ *K* ^21^_2_ *M* ^1^_2_ CF. Note, that 11 of the 12 previously reported zinc(II) complexes with bipy, bpa or bpe and malonate derivatives have the same CF and form as the **ins** 3,4-coordinated net (*e.g.* it contains two nodes, one node is the anion connected with three metal atoms, and the four-coordinated node is zinc connected with four neighbours – here, three anions and one zinc atom through a bridge *L*). The ideal **ins** net occurs in the orthorhombic *Pnnm* space group with the longest crystal parameter twice the length of the shortest one. Among the 11 reported and the new zinc(II) malonate complexes containing bipy and its analogues and **ins** architecture, only [Zn_2_(bipy)(Me_2_mal)_2_(H_2_O)_2_] (**1**), [Zn_2_(bipy)(cbdc)_2_(H_2_O)_2_] [(Zhang *et al.* (2014 ▸) cbdc = cyclobutane-1,1-dicarboxylato] and [Zn_2_(bpa)(Memal)_2_(H_2_O)_2_] (Déniz *et al.*, 2012 ▸) crystallize in the same *Pnnm* space group and similar crystal parameters; thus, (**1**) can be regarded as isostructural with [Zn_2_(bipy)(cbdc)_2_(H_2_O)_2_] (Zhang *et al.*, 2014 ▸) and [Zn_2_(bpa)(Memal)_2_(H_2_O)_2_] (Déniz *et al.*, 2012 ▸). At the same time, despite the fact that other complexes crystallize in *P*2_1_/n, *Pna*2_1_ or *Pnn*2 space groups, their crystal parameters are all close to 7.2, 7.4 and 21.0 Å, respectively, and the distortion of the network accounted for by the various substituents at the malonate anion and elongation of the *L* linker is insignificant. The net can be regarded as a combination of [Zn*An*(H_2_O)] layers with the *AK* ^21^ *M* ^1^ CF and **hcb** topology, connected through *L* linkers that bridge between zinc(II) atoms from neighbouring sheets (Fig. 9 ▸). The coordination mode of the anion does not affect the Zn–Zn interatomic distances; in (**1**) these are ∼5.4 Å as in complex (**5**) described previously. The length of a linker affects only the distance between layers, the Zn–Zn distance in bipy-containing compounds is ∼11.5 Å and that in bpe and bpa-containing compounds is ∼13.5 Å. As *N*,*N*′-containing ligands are not perpendicular to the [Zn*An*(H_2_O)] layers, the distance between them is longer than 5.4 Å and varies from 7.1 to 7.5 Å. To sum up, the family of [Zn_2_ *L* *An*_2_(H_2_O)_2_] complexes forms an isoreticular series for a variety of ligands [*L* = bipy, bpe, bpa and 4,4′-(propane-1,3-diyl)bipyridine; *An* = mal, Memal, Me_2_mal, cyclobutane­dicarboxylate (cbdc)].

**Figure 10:**
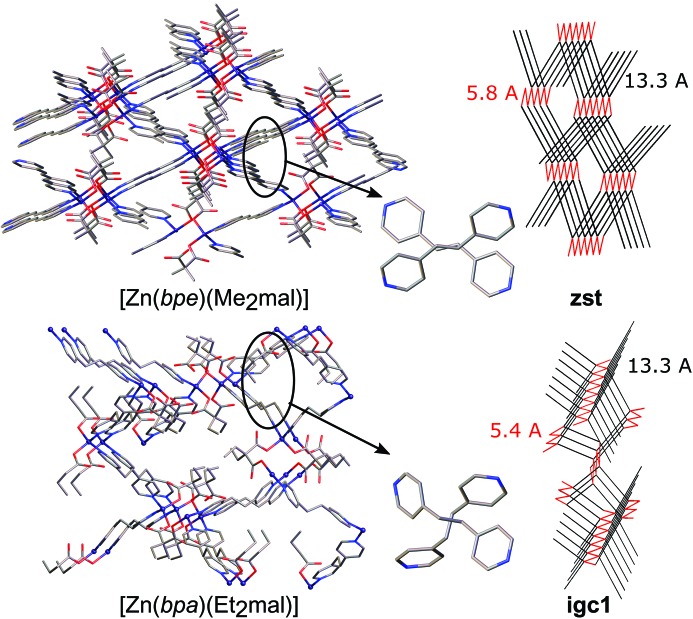
Fragment of the crystal packing in [Zn(bpe)(Me_2_mal)]·H_2_O (**2**) and [Zn(bpa)(Et_2_mal)]·0.38H_2_O (**9**) and their underlying nets depicted in black (for bpa and bpe linkers) and red (for anions). Hydrogen atoms have beem omitted.

At the same time, reactions of zinc(II) acetate with Me_2_mal or Et_2_mal in the presence of bpe and bpa, but not bipy, afforded complexes [Zn(bpe)(Me_2_mal)]·H_2_O (**2**) and [Zn(bpa)(Me_2_mal)]·H_2_O (**4**) or [Zn(bpe)(Et_2_mal)] (**7**) and [Zn(bpa)(Et_2_mal)]·0.38H_2_O (**9**), having the same CF. As the CF *AB* ^2^ *B* ^2^ allows a variety of architectures, similar networks were expected only for isostructural (**2**) and (**4**). Indeed, these realize the **zst** topology of the underlying net which can be regarded as a parallel packing of zigzag [Zn*An*] chains connected by neutral bpe or bpa linkers with water molecules situated in the pores of the net (Fig. 10 ▸). Again, the anions fix the distance between zinc(II) atoms within the [Zn*An*] chains as short as 5.8 Å; the length of a neutral linker defines the distance between the chains (13.3 Å). The fact that the linkers are not parallel allows rather tight packing of nitrogen-containing ligands so that a [2 + 2] cycloaddition reaction successfully occurred in (**2**) (see §2.4). In [Zn(bpa)(Et_2_mal)] (**9**) the same parallel zigzag [Zn*An*] chains with *r*(Zn–Zn) = 5.4 Å are revealed, which are connected by bridging bpa ligands, but the disposition of the bpa molecules in the network differs from that in the **zst** net (Fig. 10 ▸). In fact, we synthesized a new, previously unreported 4-*c* net with the point symbol (6^5^.8), registered at *ToposPro* TTD collection as **igc1** (see supporting information for *Systre* input files for this, and all the other nets).

Complex [Zn(bpe)(Et_2_mal)]·0.25bpe (**7**) crystallizes in the *Cmcm* space group for which automatic determination of net topology is restricted by the presence of numerous symmetry elements [for example, zinc(II) is eight-coordinated with all positions only half-occupied]. Careful analysis of the net assuming *P*1 symmetry allowed us to restore connectivity of a network for a coordination polymer with the [Zn(bpe)(Et_2_mal)] composition, zinc(II) in tetrahedral ZnN_2_O_2_ environment and bridge bpe and Et_2_mal linkers, and to estimate net architecture. Surprisingly, (**7**) also has a 4-coordinated (6^5^.8) topology very similar with **igc1**, but the number of zinc(II) atoms in the fifth and other coordination spheres differs from that in **igc1**. Close disposition of coordinated and co-crystallized bpe ligands allows their packing in reactive positions, although strong disorder does not allow prediction of which of the two tpcb stereoisomers can be obtained in this crystal.

Thus, eight of the nine novel compounds are characterized by one of the most expected compositions and ligand connectivities, and seven also have one of the highly probable topologies of the underlying net. This means that previously reported crystallographic data can be used to assume the most abundant architectures of complexes crystallized in complex reaction mixtures. It was demonstrated that bpe and bpa ligands in zinc(II) complexes with malonate derivatives form isoreticular series more readily than their bipy analogues. Initially, these results appear to contradict the results obtained by Kim *et al.* (2017 ▸), who studied zinc(II) alkane (or alkene)-dicarboxylates with bpe and bpa and found only few isoreticular compounds. But the absence of isoreticularity was associated with various coordination modes of anions; so for dicarboxylate anions which have only a small number of coordination modes, one can expect isoreticular complexes with bpa and bpe.

Regarding the effects of the zinc(II) precursors, one can propose that starting from zinc(II) nitrate, zinc(II)–aqua complexes with nitrogen-containing ligands and acidic salts (which act as counterions or terminal ligands) form more readily. Conversely, using [Zn(CH_3_COO)_2_(H_2_O)_2_] as a precursor would likely produce networks constructed of [Zn*An*] chains or layers connected by nitrogen-containing linkers, probably because *An* is able to replace acetate ions in the coordination sphere of a zinc(II) atom. The other part of the coordination sphere of a zinc(II) atom can contain neutral water as well as *N*,*N*′-containing ligands. Although the distance between metal atoms in the [Zn*An*] chain is not very small (*ca* 5 Å), various types of connections between the chains sometimes allow close packing of bpe ligands in reactive positions for post-synthetic modification by means of photoinitiated reactions.

### 2.4. Single-crystal-to-single-crystal [2 + 2] cycloaddition reactions   

Three of the four bpe-containing compounds have a close disposition of the bpe ligands; these were attested to undergo a solid-state photoinitiated [2 + 2] cycloaddition reaction. Their single crystals were irradiated with a Xe lamp and then analyzed by means of single-crystal XRD. Bulk samples were investigated using powder XRD and ^1^H NMR. All three methods confirmed that compounds had undergone solid-state reactions.

#### 2.4.1. **Complex (2)**   

**Figure 11:**
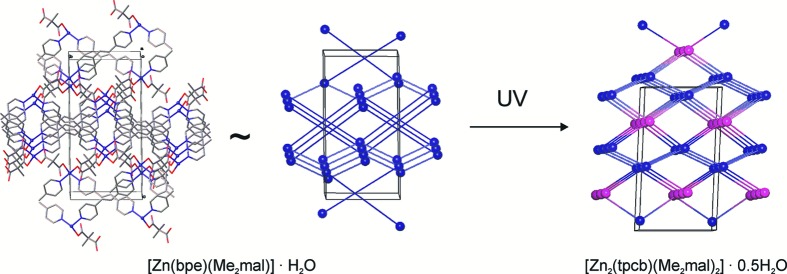
Fragment of crystal packing in [Zn(bpe)(Me_2_mal)]·H_2_O (**2**), the underlying net and the hypothetical underlying net of [Zn_2_(tpcb)(Me_2_mal)_2_]·0.5H_2_O.

**Figure 12:**
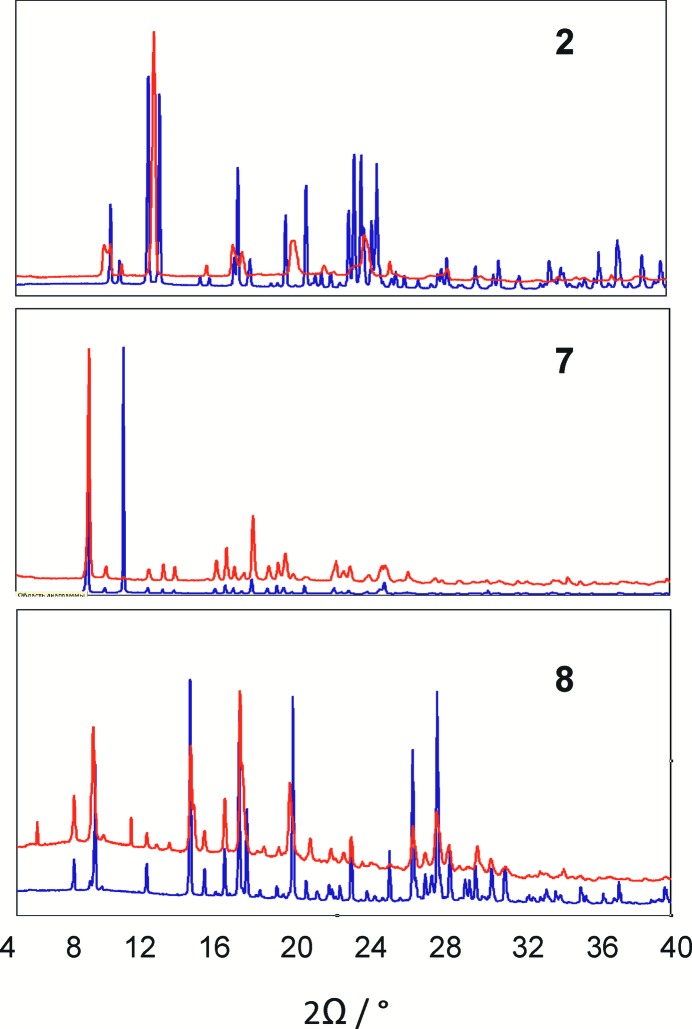
Powder XRD data for (**2**), (**7**) and (**8**) before irradiation (blue) and after irradiation for 6 h (red).

Mutual disposition of bpe ligands in (**2**) is not very efficient for a [2 + 2] cycloaddition reaction (Figs. 10, 11); neither of the two ligands are coplanar, no ethylene fragments are parallel, the distances between the carbon atoms of the ethylene groups are equal to 3.9 and 4.4 Å. At the same time, powder XRD (Fig. 12 ▸) clearly indicates that UV irradiation affects the structure and a solid-state reaction has taken place. UV irradiation of a single crystal under N_2_ flow for 4 h did not affect the structure; thus, the same single crystal was irradiated in air at room temperature for a further 8 h. In this case, reflection intensities changed dramatically, so that the structure could be obtained using synchrotron radiation only. XRD revealed the reasons for the varied behaviour of the sample at low temperatures in air. During irradiation in air, the sample was also heated and the quantity of water molecules in the crystal structure decreased. The absence of a proportion of water molecules could affect the mutual disposition of the bpe molecules in the coordination polymer and could promote the photoinitiated [2 + 2] cycloaddition reaction. Loss of solvent molecules followed by other single-crystal-to-single-crystal (SCSC) reactions was previously observed in other zinc(II) carboxylates (Ou *et al.*, 2011 ▸; Li *et al.*, 2014 ▸), so this reaction is a new example of a two-step SCSC process. The reaction proceeded with a change in space group (*P*2_1_/*c → Pnna*) and despite the loss of solvent, incurred a 1.5% increase in cell volume. The number of water molecules in the reaction product obtained by free refinement of the occupancy of a water oxygen is equal to 0.25 [per zinc(II) atom]; thus, one can hardly expect full conversion of bpe molecules. Indeed, the refinement of the occupancies of carbon atoms gives the ratio of one molecule of the *rtct* isomer of tetrakis(4-pyridyl)cyclobutane (**tpcb**) per two molecules of bpe and the composition of the complex obtained by an SCSC two-step process [Zn(bpe)(Me_2_mal)]_2_[Zn_2_(tpcb)(Me_2_mal)_2_]·H_2_O. ^1^H NMR data (Fig. S15) also provides evidence that the reaction product contains only the *rtct* isomer as proposed from the preorganization of ligands within the crystal (Fig. 10 ▸). Both ^1^H NMR and PXRD data (Fig. S6) indicate that the conversion of the bpe molecules in the sample was unfinished.

As the initial complex and reaction product are both three-periodic polymers, the photoinitiated process in (**2**) is of the 3D→3D type. At the same time, the reaction product contains the *K* ^4^ ligand instead of two *B* ^2^ ligands; thus, the change in the network is also of interest. Assuming full conversion of the polymer to [Zn_2_(tpcb)(Me_2_mal)_2_]·0.5H_2_O, we estimated the topology of the irradiated sample to be the 4,4,4-coordinated net with the point symbol {4·6^4^·8}{6^5^·8}{4^3^·6^3^} (Fig. 11 ▸). This is also a novel net that has not been seen before.

#### 2.4.2. **Complex (7)**   

**Figure 13:**
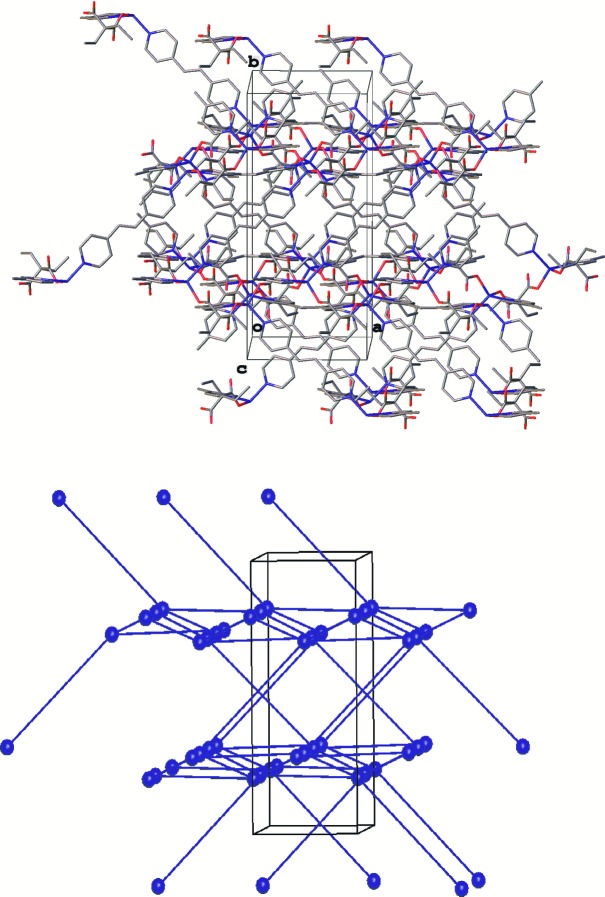
Fragment of the crystal packing in [Zn(bpe)(Et_2_mal)]·0.25bpe (**7**) (top) and its underlying net (bottom).

A crystal of (**7**) underwent a *Cmcm → Pbcn* transfer in an SCSC manner after irradiation for 6 h with a 0.9% decrease in cell volume. PXRD indicates total conversion of the bulk sample; ^1^H NMR revealed that the sample contains both *rctt*-tetrakis(4-pyridyl)cyclobutane as the major reaction product and trace amounts (<5%) of the *rtct* isomer (Fig. S16). Single-crystal XRD of the irradiated crystal showed that the reaction product is fully occupied and ordered in contrast with the initial compound, and indeed contains a 3D coordination polymer with the [Zn(bpe)_0.75_(tpcb)_0.25_(Et_2_mal)] composition, the *AB* ^2^ *B* ^2^ CF and the net topology proposed for (**7**) (registered as **igc2**). The net is depicted in Fig. 13 ▸; it can also be described, again, as parallel packing of the [Zn(Et_2_mal)] zigzag chains connected to **hcb** layers by one of the two independent bpe molecules. These layers are further connected to the **igc2** net by means of bridge ligands (these positions are shared with bpe and tpcb molecules). Only XRD analysis of the reaction product allowed us to undoubtedly reveal the presence of co-crystallized bpe molecules in the pores of the initial complex (**7**), because these are highly disordered in the *Cmcm* space group. The disorder does not allow the mutual disposition of coordinated and co-crystallized bpe molecules to be confirmed and hence an estimation of which of the two tpcb isomers should form more readily. But based on ^1^H NMR data, one can propose that there exists some pedal-like motion prior to photodimerization, as both isomers appear in this spectrum (but only the *rctt* isomer was found as a single crystal). As the reaction occurred between the coordinated and co-crystallized bpe molecules, the coordination network remained unchanged.

#### 2.4.3. **Complex (8)**   

**Figure 14:**
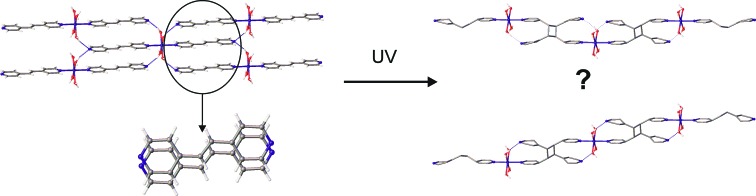
H-bonded layers in (**8**) and possible coordination polymers obtained after UV irradiation of a single crystal of (**8**).

The parallel orientations of the discrete [Zn(bpe)_2_(H_2_O)_4_]^2+^ complexes in (**8**) are stabilized by intermolecular O—H⋯N bonds (Fig. 14 ▸). The mean planes of bpe ligands are parallel and the distance between equivalent atoms is equal to 3.7366 (3) Å. Being constructed from 1,4-bis(pyridin-4-yl)buta-1,3-diene or 1,4-bis(pyridin-4-yl)buta-1,3-diyne, such architectures could give a 0D→2D photoreaction with metal–organopolymeric coordination networks. In this case only a 0D→1D [2 + 2] cycloaddition occurred during UV irradiation. Mutual disposition of bpe ligands in (**8**) allows the *rctt* tpcb to act as a bridge ligand, and the [Zn_2_(tpcb)(H_2_O)_8_]^4+^ chain is the only reaction product. The reaction proceeds in an SCSC manner so that the resulting structure is also suitable for single-crystal XRD. XRD data indicates that after irradiation for 3 h the single crystal contained 15% of initial substance. Further irradiation of the same single crystal does not change the conversion rate. The geometry of the chain (linear, zigzag or random, see Fig. 14 ▸) can not be evaluated, because the cyclobutane ring is probably equally disordered over two sites by symmetry. The presence of both bpe and *rctt* tpcb molecules was also demonstrated by powder XRD (Fig. S13) and ^1^H NMR data (Fig. S17).

To sum up, we succeeded in carrying out the [2 + 2] cycloaddition reactions in an SCSC manner for three of the four novel compounds obtained from Zn^II^:bpe:Me_2_mal (or Et_2_mal):H_2_O mixtures.

## 3. Conclusions   

We analyzed the crystal structures of previously reported zinc(II) complexes with *N*,*N*′-containing bidentate linkers in order to establish the possible coordination architectures of zinc(II) malonate complexes with 1,2-bis(pyridin-4-yl)ethylene and its photo-insensitive analogues. Nine novel compounds obtained from Zn^II^:bpe:Me_2_mal(or Et_2_mal):H_2_O mixtures have highly probable compositions, zinc(II) environments and ligand connectivities, estimated from previously reported crystallographic data for zinc(II) complexes. Seven complexes also have one of the highly expected topologies of underlying nets and two form novel networks. A series of isoreticular coordination polymers containing malonate derivatives and analogues of 4,4′-bipyridine were found. 1,2-Bis(pyridin-4-yl)ethane was found to be a better analogue of 1,2-bis(pyridin-4-yl)ethylene than 4,4′-bipyridine. We found that zinc(II) acetate readily forms the target 2D and 3D architectures, whereas zinc(II) nitrate solutions produced aqua complexes and complexes with acidic malonates. Replacement of the acetate groups in the zinc(II) coordination sphere will most likely result in the formation of neutral [Zn*An*] architectures with relatively short Zn^II^–Zn^II^ distances, followed by the interaction with nitrogen-containing linkers. As these linkers are longer than bridging anions, the [Zn*An*] chains can be connected by neutral linkers in numerous ways to obtain polymers with parallel or interwoven orientation of the latter.

Three of the four novel complexes containing 1,2-bis(pyridin-4-yl)ethylene were found to have close packing of ethylene groups (<4.2 Å). We succeeded in carrying out a photoinitiated [2 + 2] cycloaddition reaction for these compounds in an SCSC manner. The UV irradiation of the crystals of {[Zn(bpe)(Me_2_mal)]·H_2_O}*_n_*, {[Zn(bpe)(Et_2_mal)]·0.25bpe}*_n_* and [Zn(H_2_O)_4_(bpe)_2_](HEt_2_mal)_2_ afforded the 3D→3D (with network change), 3D→3D (with a constant net topology) and 0D→1D transformations with formation of {[Zn(bpe)(Me_2_mal)]_2_[Zn_2_(tpcb)(Me_2_mal)_2_]·H_2_O}*_n_*, [Zn(bpe)_0.75_(tpcb)_0.25_(Et_2_mal)]_*n*_ and {[Zn(H_2_O)_4_(bpe)_2_]_0.15_[Zn(H_2_O)_4_(tpcb)]_0.85_(HEt_2_mal)_4_}_*n*_, respectively. Overall, we think that our study provides a foundation for future development in the application of reticular synthesis as well as a big data analysis for a synthetic approach to photoreactive coordination polymers.

## Supplementary Material

Crystal structure: contains datablock(s) 1, 2, 2a, 3, 4, 5, 6, 7, 7a, 8, 8a, 9. DOI: 10.1107/S2052252518001641/lq5010sup1.cif

Structure factors: contains datablock(s) 1. DOI: 10.1107/S2052252518001641/lq50101sup2.hkl

Structure factors: contains datablock(s) 2. DOI: 10.1107/S2052252518001641/lq50102sup3.hkl

Structure factors: contains datablock(s) 2a. DOI: 10.1107/S2052252518001641/lq50102asup4.hkl

Structure factors: contains datablock(s) 4. DOI: 10.1107/S2052252518001641/lq50104sup5.hkl

Structure factors: contains datablock(s) 5. DOI: 10.1107/S2052252518001641/lq50105sup6.hkl

Structure factors: contains datablock(s) 6. DOI: 10.1107/S2052252518001641/lq50106sup7.hkl

Structure factors: contains datablock(s) 7. DOI: 10.1107/S2052252518001641/lq50107sup8.hkl

Structure factors: contains datablock(s) 7a. DOI: 10.1107/S2052252518001641/lq50107asup9.hkl

Structure factors: contains datablock(s) 8. DOI: 10.1107/S2052252518001641/lq50108sup10.hkl

Structure factors: contains datablock(s) 8a. DOI: 10.1107/S2052252518001641/lq50108asup11.hkl

Structure factors: contains datablock(s) 9. DOI: 10.1107/S2052252518001641/lq50109sup12.hkl

Supporting figures and tables. DOI: 10.1107/S2052252518001641/lq5010sup13.pdf

CCDC references: 1568619, 1568620, 1568621, 1568622, 1568623, 1568624, 1568625, 1568626, 1568627, 1568628, 1568629, 1568630

## Figures and Tables

**Figure 4:**
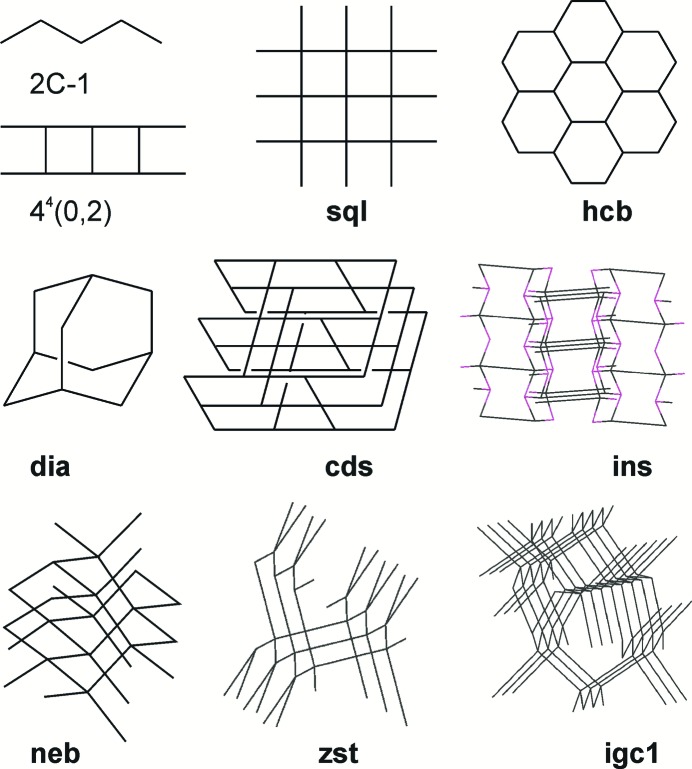
Selected 1D, 2D and 3D underlying nets derived for zinc(II) malonates.
