# Supplementary material for: Exploitation of knowledge databases in the synthesis of zinc(II) malonates with photo-sensitive and photo-insensitive N,N′-containing linkers
Source: IUCrJ. 2018 Mar 27;5(Pt 3):293–303. doi: 10.1107/S2052252518001641 (PMC5929375; doi:10.1107/S2052252518001641)
Supplement: Supplementary file 13 [file m-05-00293-sup13.pdf]

IUCrJ

Volume 5 (2018)

Supporting information for article:

Exploitation of knowledge databases in the synthesis of zinc(II) malonates with photo-sensitive and photo-insensitive *N*,*N*′-containing linkers

Ekaterina N. Zorina-Tikhonova, Aleksandr S. Chistyakov, Mikhail A. Kiskin, Aleksei A. Sidorov, Pavel V. Dorovatovskii, Yan V. Zubavichus, Eugenia D. Voronova, Ivan A. Godovikov, Alexander A. Korlyukov, Igor L. Eremenko and Anna V. Vologzhanina

Table S1 Previously Reported Zinc(II) Malonate Complexes with 4,4'-Bipyridine and its Analogs.

Complex[a]	L : Zn	CCF	net	Refcode
$[Zn_2$(*bipy*)(Memal)$_2(H_2O)_2]$	0.5	$A_2B^2K^{21}{}_2M^1{}_2$	**ins**	GAKVEV
$[Zn_2$(*bipy*)(cbdc)$_2(H_2O)_2]$	0.5	$A_2B^2K^{21}{}_2M^1{}_2$	**ins**	IHEFIM
$[Zn_2$(*bipy*)(cbdc)$_2(H_2O)_2]$	0.5	$A_2B^2K^{21}{}_2M^1{}_2$	**ins**	IHEFIM01
$[Zn_2$(*bipy*)(mal)$_2(H_2O)_2]$ · $0.5H_2O$	0.5	$A_2B^2K^{21}{}_2M^1{}_2$	**ins**	JIQKIE
$[Zn_2$(*bipy*)(mal)$_2(H_2O)_2]$ · MeCN · H_2O	0.5	$A_2B^2K^{21}{}_2M^1{}_2$	**ins**	KEXKAB
$[Zn_2$(*pdd*)(mal)$_2(H_2O)_2]$ · 2MeCN	0.5	$A_2B^2K^{21}{}_2M^1{}_2$	**ins**	KEXKEF
$[Zn_2$(*bpe*)(mal)$_2(H_2O)_2]$ · 2MeCN	0.5	$A_2B^2K^{21}{}_2M^1{}_2$	**ins**	KEXPIO
$[Zn_2$(*bpa*)(mal)$_2(H_2O)_2]$ · 2MeCN	0.5	$A_2B^2K^{21}{}_2M^1{}_2$	**ins**	KEXPOU
$[Zn_2$(*bpe*)(Memal)$_2(H_2O)_2]$	0.5	$A_2B^2K^{21}{}_2M^1{}_2$	**ins**	TEMTIQ
$[Zn_2$(*bpa*)(Memal)$_2(H_2O)_2]$	0.5	$A_2B^2K^{21}{}_2M^1{}_2$	**ins**	TEMTOW
$[Zn_2$(*bpa*)(cbdc)$_2(H_2O)_2]$	0.5	$A_2B^2K^{21}{}_2M^1{}_2$	**ins**	YAMYIX
[Zn(*bipy*)(Ph$(CH_2)_3$mal)$_2(H_2O)_2]$ · $4H_2O$	1	$AB^2T^{11}M^1$	**sql**	ZEQJAI

[a] Memal = methylmalonate; cbcd = cyclobutane-1,1-dicarboxylate; mal = malonate; pdd = 4,4'-(propane-1,3-diyl)dipyridine.

Table S2 The most probable complexes and their coordination formulas and networks in the Zn^{II} : An^{2-} (or HAn^{-}) : *L* : H_2O mixtures

Ratio - ratio of L and Zn^{II}; CN - coordination number of Zn^{II}; CP - coordination polyhedron; CF - coordination formula; possible nets are taken from previously reported databased of networks obtained for coordination polymers [S1-S3] or taken for a database of zinc(II) complexes with *bipy*, *bpe* or *bpa*.

Ratio L:Zn^{II}	CN	CP	CF	Complex	nets	Examples
0.5	4	NO_3	$A_2B^2M^1{}_6$	$[Zn_2L(H_2O)_6]^{4+}$	dimer	-
			$A_2B^2B'^2{}_2M^1{}_2$	$[Zn_2LAn_2(H_2O)_2]$	2C1 (1D), $4^4(0,2)$ (1D) **hcb** (2D), **sql** (2D), 3,3L5 (2D), 3,3,3T9 (3D), **etb** (3D), **srs** (3D)	$[Zn_2(bpe)(Et_2mal)_2(bpe)_2]$ (**5**) - **hcb**; {CIXNAZ} - **hcb**; {KEBTER} - $4^4(0,2)$; {LEDLAJ} - **srs**; {LEVPAF} - **etb**; {MENMAU} - $4^4(0,2)$; {PIXWID} $4^4(0,2)$; {TOFLOR} - 2C1; {TUBBAV} - **hcb**; {VIXVIJ} - **hcb**; {UNAWUC} **hcb**; {WAJGEU} - **hcb**; {XIZLIC} - $4^4(0,2)$; {YOQMUO} - **hcb**.
			$A_2B^2B^{01}{}_2M^1{}_2$		dimer	-
			$A_2B^2B^{11}{}_2$	$[Zn_2LAn_2]$	2C1 (1D); $4^4(0,2)$ (1D)	{UYASOD} - $4^4(0,2)$
			$A_2B^2T^{11}{}_2$		**hcb** (2D), **sql** (2D), lpx1	-
	5	NO_4	$A_2B^2M^1{}_8$	$[Zn_2L(H_2O)_8]^{4+}$	dimer	-
			$A_2B^2B'^2{}_2M^1{}_4$	$[Zn_2LAn_2(H_2O)_4]$	2C1 (1D), $4^4(0,2)$ (1D) **hcb** (2D), **sql** (2D), 3,3L5 (2D), 3,3,3T9 (3D), **etb** (3D)	{IJUXUG} - $4^4(0,2)$.
			$A_2B^2B^{01}{}_2M^1{}_4$		dimer	-
			$A_2B^2B^{11}{}_2M^1{}_2$	$[Zn_2LAn_2(H_2O)_2]$	2C1 (1D), $4^4(0,2)$ (1D)	-
			$A_2B^2T^{11}{}_2M^1{}_2$		**hcb** (2D), **sql** (2D), lpx1	{VIXVIJ} - **hcb.**
			$A_2B^2T^{21}{}_2$	$[Zn_2LAn_2]$	3,4L83	-
			$A_2B^2K^{21}{}_2$		3,4L83 3,4L95, 3,4L101 3,4L112 , **fsc** (3D), **ins** (3D), 3,4T10 (3D)	-
	6	NO_5	$A_2B^2M^1{}_{10}$	$[Zn_2L(H_2O)_{10}]^{4+}$	dimer	-
			$A_2B^2B'^2{}_2M^1{}_6$	$[Zn_2LAn_2(H_2O)_6]$	2C1 (1D), $4^4(0,2)$ (1D) **hcb** (2D), **sql** (2D), 3,3L5 (2D), 3,3,3T9 (3D), **etb** (3D)	-
			$A_2B^2B^{01}{}_2M^1{}_6$		dimer	-
			$A_2B^2B^{11}{}_2M^1{}_4$	$[Zn_2LAn_2(H_2O)_4]$	2C1 (1D), $4^4(0,2)$ (1D)	-

			$A_2B^2T^{11}{}_2M^1{}_4$		**hcb** (2D), **sql** (2D), lpx1	-
			$A_2B^2T^{21}{}_2M^1{}_2$	$[Zn_2LAn_2(H_2O)_2]$	3,4L83	-
			$A_2B^2K^{21}{}_2M^1{}_2$		3,4L83 3,4L95, 3,4L101 3,4L112 , **fsc** (3D), **ins** (3D), 3,4T10 (3D)	[Zn$_2$(*bipy*)(Me$_2$mal)$_2$(H$_2$O)$_2$] (**1**); {GAKVEV} {IHEFIM} {JIQKIE} {KEXKAB} {KEXKEF} {KEXPIO}; {KEXPOU} {TEMTIQ} {TEMTOW} {YAMYIX} - **ins** for all
1	4	NO_3	$AM^1M'^1{}_3$	$[ZnL(H_2O)_3]^{2+}$	0D	-
			$AM^1B^2M'^1$	$[ZnLAn(H_2O)]$	dimer, 2C1 (1D)	-
			$AM^1B^{01}M'^1$		0D	-
			AM^1B^{11}	[ZnLAn]	dimer	-
			AM^1T^{11}		dimer, tetramer, 2C1 (1D)	-
		N_2O_2	$AB^2M^1{}_2$	$[ZnL(H_2O)_2]$ $[ZnL(HAn)_2]$	dimer, 2C1 (1D)	[Zn(*bpe*)(HMe$_2$mal)$_2$] (**3**); {DAYCEM} {DAZYEI} {EBITEQ} {EDUNOH} {FADWAK} {FIFXAU} {GOLTAD} {GOXKUZ} {GOYRAN} {HUVJOZ} {ICOVAZ} {IXAJAT} {IXOTAR} {JESRUV} {JOKKIF} {JOMWUF} {LIGYUX} {MAHROF} {MOPNIQ} {MOYGIR} {MUFTOX} {MUFTUD} {MUZNOM} {PASNOO} {PIHKEX} {REFLAR} {RILWIU} {SENKAY} {SUSWAF} {TEGTII} {TEGTII} {TUSKUN} {TUSLAU} {UBOCIX} {UBOCOD} {UBUTUI} {VIYQAX} {XOBWIV} {ZAWREW} {ZAWRIA} {ZAZDAH} {CASHOU} - 2C1 for all
			$AB^2B'^2$	[ZnLAn]	$4^4(0,2)$ (1D), **sql** (2D), **kgl** (2D), 4L1 (2D), **dia**(3D), **pts** (3D), **lon** (3D), **dmp** (3D), **qtz** (3D), **cds** (3D), **uoc** (3D), 4T12 (3D), **mmt** (3D), **neb** (3D), **nbo** (3D)	[Zn(*bpe*)(Me$_2$mal)] (**2**) - **zst**; [Zn(*bpa*)(Me$_2$mal)] (**4**) - **zst**, [Zn(*bpe*)(Et$_2$mal)]·0.5*bpe* (**7**) - **igc2**; [Zn(*bpe*)$_{0.75}$(tpcb)$_{0.25}$(Et$_2$mal)] (**7a**) - **igc2**; [Zn(*bpa*)(Et$_2$mal)] (**9**) - **igc1**; {CUWGAD} - **sql**; {CUYKEN} - **dia**; {IWEPEF} - **dia**; {IWEPIJ} - **dia**; {UHOMIP} - **cds**; {VAJTEH} - **neb**; {ZARZAV} - **dia**
			AB^2B^{01}		dimer, 2C1 (1D)	-
	5	NO_4	$AM^1M'^1{}_4$	$[ZnL(H_2O)_4]^{2+}$	0D	-
			$AM^1B^2M'^1{}_2$	$[ZnLAn(H_2O)_2]$	dimer, 2C1 (1D)	-
			$AM^1B^{01}M'^1{}_2$		0D	-
			$AM^1B^{11}M^1$	$[ZnLAn(H_2O)]$	dimer	-
			$AM^1T^{11}M^1$		dimer, tetramer, 2C1 (1D)	-
			AM^1T^{21}	[ZnLAn]	**hcb** (2D), **fes** (2D)	-

		AM^1K^{21}		4^4(0,2) (1D), **hcb** (2D), **fes** (2D), 3,4L13	-
	N_2O_3	$AB^2M^1{}_3$	$[ZnL(H_2O)_3]^{2+}$	dimer, 2C1 (1D)	-
		$AB^2B'^2M^1$	$[ZnLAn(H_2O)]$	4^4(0,2) (1D), **sql** (2D), **kgl** (2D), 4L1 (2D), **dia**(3D), **pts** (3D), **lon** (3D), **dmp** (3D), **qtz** (3D), **cds** (3D), **uoc** (3D), 4T12 (3D), **mmt** (3D), **neb** (3D), **nbo** (3D)	{OPUTEZ} - **sql**
		$AB^2B^{01}M^1$		dimer, 2C1 (1D)	-
		AB^2B^{11}	[ZnLAn]	**sql** (2D), 4L1 (2D), 4L2 (2D)	-
		AB^2T^{11}		**sql** (2D), 4L1 (2D), 4L2 (2D)	-
6	NO_5	$AM^1M'^1{}_5$	$[ZnL(H_2O)_5]^{2+}$	0D	-
		$AM^1B^2M'^1{}_3$	$[ZnLAn(H_2O)_3]$	dimer, 2C1 (1D)	-
		$AM^1B^{01}M'^1{}_3$		0D	-
		$AM^1B^{11}M^1{}_2$	$[ZnLAn(H_2O)_2]$	Dimer	-
		$AM^1T^{11}M^1{}_2$		dimer, tetramer, 2C1 (1D)	-
		$AM^1T^{21}M^1$	$[ZnLAn(H_2O)]$	**hcb** (2D), **fes** (2D)	-
		$AM^1K^{21}M^1$		4^4(0,2) (1D), **hcb** (2D), **fes** (2D), 3,4L13	-
	N_2O_4	$AB^2M^1{}_4$	$[ZnL(H_2O)_4]^{2+}$	dimer, 2C1 (1D)	$[Zn(H_2O)_4($*bipy*$)](HEt_2mal)_2$ (**6**) {AKIXEX} {BUYYIE} {DITLOI} {EKOFOA} {EHITOG} {EXUMUG} {FAXLIA } {GALHIM} {GUFHAR } {HUCJIZ } {IQUQOC } {JEKRUN } {JESQEE } {KETGEX } {NASZOW } {NASZUC } {NATBAL } {NIJTOP } {POWFOX } {QOTSIC } {ROBQUV} {SORDUZ } {TOLJOU } {TULKUH } {XIYPOL } {YARJUY} {YOQJIZ }- 2C1 for all
		$AB^2B'^2M^1{}_2$	$[ZnLAn(H_2O)_2]$	4^4(0,2) (1D), **sql** (2D), **kgl** (2D), 4L1 (2D), **dia**(3D), **pts** (3D), **lon** (3D), **dmp** (3D), **qtz** (3D), **cds** (3D), **uoc** (3D), 4T12 (3D), **mmt** (3D),	-

					**neb** (3D), **nbo** (3D)	
			$AB^2B^{01}M^1_2$		dimer, 2C1 (1D)	-
			$AB^2B^{11}M^1$	$[ZnLAn(H_2O)]$	**sql** (2D), 4L1 (2D), 4L2 (2D)	-
			$AB^2T^{11}M^1$		**sql** (2D), 4L1 (2D), 4L2 (2D)	{ZEQJAI} - **sql**
			AB^2T^{21}	$[ZnLAn]$	3,4L83, 3,5L2, gek1	-
			AB^2K^{21}		3,5L2 3,4L83 4^4(0,4) (1D), 4^4(1,4) (1D), **sql** (2D), **dia** (3D), **neb-e** (3D) **fet** (3D)	{SUJQOE} - **fet**; {SUJQUK} - **fet**
2	4	N_2O_2	$AM^1_2M'^1_2$	$[ZnL_2(H_2O)_2]^{2+}$ $[ZnL_2(HAn)_2]$	0D	-
			$AM^1_2B^2$	$[ZnL_2An]$	dimer, 2C1 (1D)	-
			$AM^1_2B^{01}$		0D	-
		N_4	AB^2_2	$[ZnL_2]^{2+}$	4^4(0,2) (1D), **sql** (2D), **kgl** (2D), 4L1 (2D), **dia**(3D), **pts** (3D), **lon** (3D), **dmp** (3D), **qtz** (3D), **cds** (3D), **uoc** (3D), 4T12 (3D), **mmt** (3D), **neb** (3D), **nbo** (3D)	-
	5	N_2O_3	$AM^1_2M'^1_3$	$[ZnL_2(H_2O)_3]^{2+}$ $[ZnL_2(HAn)_2(H_2O)]$	0D	-
			$AM^1_2B^2M'^1$	$[ZnL_2An(H_2O)]$	dimer, 2C1 (1D)	-
			$AM^1_2B^{01}M'^1$		0D	-
			$AM^1_2B^{11}$	$[ZnL_2An]$	Dimer	-
			$AM^1_2T^{11}$		dimer, tetramer, 2C1 (1D)	-
		N_4O	$AB^2_2M^1$	$[ZnL_2(H_2O)]^{2+}$	4^4(0,2) (1D), **sql** (2D), **kgl** (2D), 4L1 (2D), **dia**(3D), **pts** (3D), **lon** (3D), **dmp** (3D), **qtz** (3D), **cds** (3D), **uoc** (3D), 4T12 (3D), **mmt** (3D), **neb** (3D), **nbo** (3D)	-
	6	N_2O_4	$AM^1_2M'^1_4$	$[ZnL_2(H_2O)_4]^{2+}$ $[ZnL_2(HAn)_2(H_2O)_2]$	0D	$[Zn(H_2O)_4(bpe)_2](HEt_2mal)_2$ (**8**) {ESIXEL} {CERFIP} {DOFWEB} {GASWAA} {GATXIJ} {KESNIH}

	$AM^{1}_{2}B^{2}M'^{1}_{2}$	$[ZnL_2An(H_2O)_2]$	dimer, 2C1 (1D)	{KUMDON} {MITXIY} {NISDEA} {SOCNOO} {UPUFUH} {VUFTOG} {ZAWJOY} {ZERPUJ} {XADVAB} - dimer
	$AM^{1}_{2}B^{01}M'^{1}_{2}$		0D	-
	$AM^{1}_{2}B^{11}M'^{1}$	$[ZnL_2An(H_2O)]$	dimer	-
	$AM^{1}_{2}T^{11}M'^{1}$		dimer, tetramer, 2C1 (1D)	-
	$AM^{1}_{2}T^{21}$	$[ZnL_2An]$	**hcb** (2D), **fes** (2D)	-
	$AM^{1}_{2}K^{21}$		4^4(0,2) (1D), **hcb** (2D), **fes** (2D), 3,4L13	-
N_4O_2	$AB^{2}_{2}M^{1}_{2}$	$[ZnL_2(H_2O)_2]^{2+}$	4^4(0,2) (1D), **sql** (2D), **kgl** (2D), 4L1 (2D), **dia**(3D), **pts** (3D), **lon** (3D), **dmp** (3D), **qtz** (3D), **cds** (3D), **uoc** (3D), 4T12 (3D), **mmt** (3D), **neb** (3D), **nbo** (3D)	-
	$AB^{2}_{2}B'^{2}$	$[ZnL_2An]$	2C-1 (1D) **sql** (2D) **pcu** (3D) **jsm** (3D)	-
	$AB^{2}_{2}B^{01}$	$[ZnL_2An]$	4^4(0,2) (1D), **sql** (2D), **kgl** (2D), 4L1 (2D), **dia**(3D), **pts** (3D), **lon** (3D), **dmp** (3D), **qtz** (3D), **cds** (3D), **uoc** (3D), 4T12 (3D), **mmt** (3D), **neb** (3D), **nbo** (3D)	-

S1. Experimental

S1.1. Coordination Formulas and Their Applications

Let us denote mono-, bi-, tri- or tetradenate ligands with M, B, T or K letters. The way in which metal atoms A surround the ligand is denoted by numerical superscripts (mbtk). The superscripts define the ‘partial’denticity of the ligand with respect to any A atom (m – mono-, b – bi-, t – tri-, k – tetradenticity). The number of A atoms with respect to the ligand that exhibits the corresponding partial denticity is denoted by the numerical value of the corresponding superscript. Then the coordination type of an i-th ligand is given as $D^{mbtk}{}_{i}$. A few examples of tridentate ligands coordinated by one, two or three metal atoms are given in Fig. S1, as well as the corresponding coordination-type symbols.

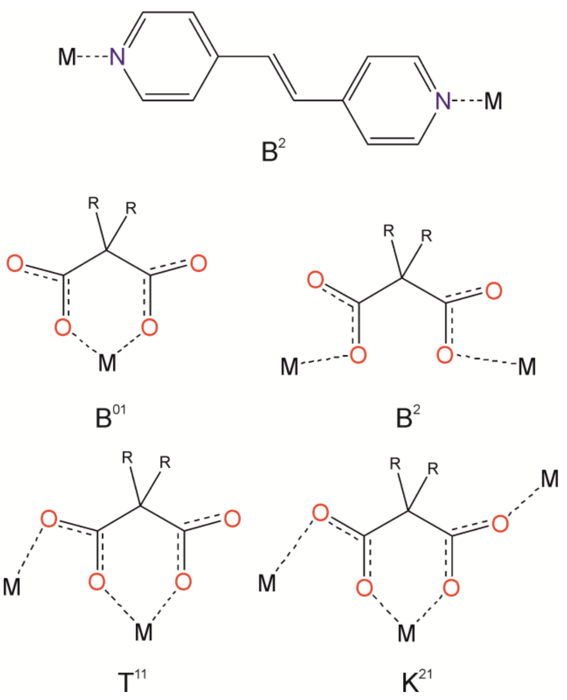

Figure S1 Selected coordination modes of *bpe* or malonate anion and its derivatives in zinc(II) complexes.

The symbol for the ligand coordination type also denotes the total number of complexing atoms (Z) which surround the ligand, and the total number of chemical bonds that the ligand makes with the central atom (N_B) as $Z = m + b + t + k$ and $N_B = 1m + 2b + 3t + 4k$.

For example, for a chelate ligand B^{01} $Z = 1\ (0 + 1)$ and $N_B = 2\ (1{\cdot}0 + 3{\cdot}1)$, while for a bridge-chelate K^{21} ligand $Z = 3\ (2 + 1)$ and $N_B = 4\ (1{\cdot}2+2{\cdot}1)$ (Fig. S1).

Provided that the coordination types of all the ligands in a complex are determined, the coordination formula (CF) of the complex can be written. Any CF includes the coordination types of all the ligands with the same chemical formula (with the exception of counterions and molecules). The subscripts denote the stoichiometric composition with respect to any equivalent ligand and a metal A atom. Using the chemical and crystallochemical formulae of a complex together allows the environment of the central atom to be

characterized in order to calculate the coordination number (CN) and the number of ligands in the first coordination sphere (N_A) without any diagramatical or text description.

$$\mathrm{CN(A)} = \sum_i \nu_i (m+2b+3t+4k)_i \quad \mathrm{N_A} = \sum_i \nu_i (m+b+t+k)_i$$

For example, a complex with composition $[ZnLAn(H_2O)_2]$ may have CF $AM^1B^2M'^1_2$ and $AM^1B^{11}M'^1_2$ if L acts as monodentate ligand, and An is bridge or bridge-chelate ligand, or $AB^2B'^2M^1_2$ if both L and An are bridge ligands. Calculation of CN and CP as possible diagramatical representations of corresponding architectures are given in Scheme S1.

	CF	CN(Zn)	CP
$[ZnLAn(H_2O)_2]$	$AM^1B^2M'^1_2$	1 + 2 + 1·2 = 5	NO_4
	$AM^1B^{11}M'^1_2$	1 + (1·1 + 1·2) + 1·2 = 6	NO_5
	$AB^2B'^2M^1_2$	2 + 2 + 1·2 = 6	N_2O_4

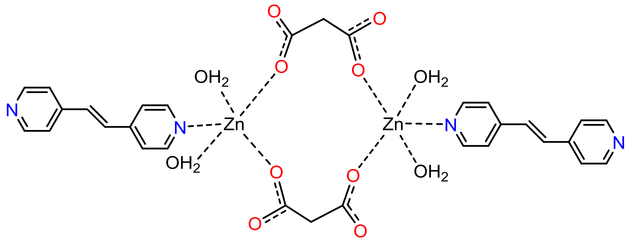

dimer

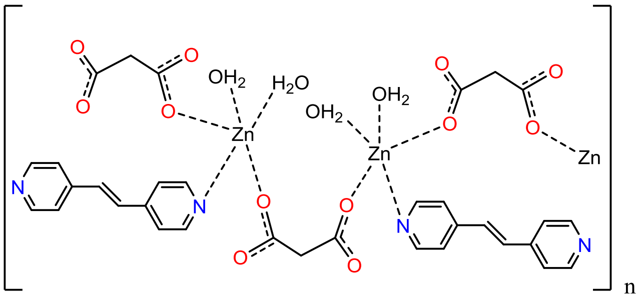

Chain 2C-1

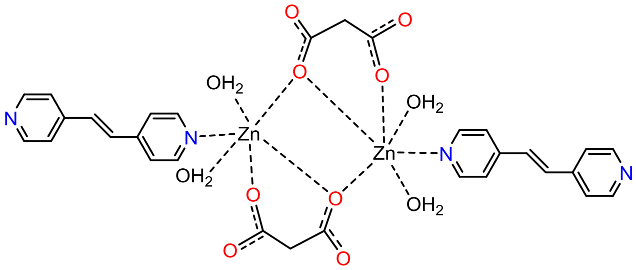

dimer

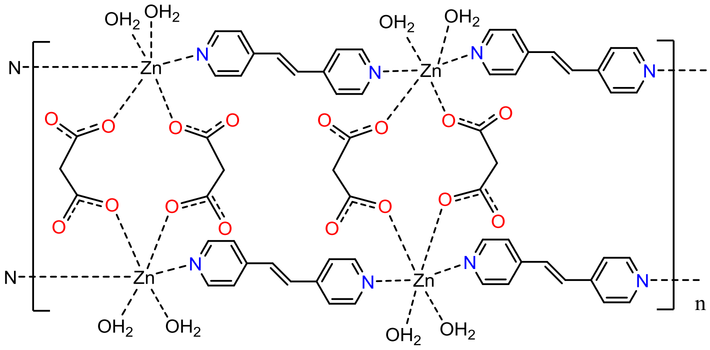

Ladder, $4^4(0,2)$

Scheme S1. Some coordination formulas for a complex with composition $[ZnLAn(H_2O)_2]$, calculation of coordination number of central atom, its polyhedron and possible architectures.

S1.2. Analysis of Previously Reported Zinc(II) Complexes with *bipy*, *bpe* and *bpa*.

Distribution of zinc(II) coordination numbers and composition of coordination polyhedra has been calculated for 1473 complexes containing both zinc(II) and at least one of *bipy*, *bpe* or *bpa* ligands. Distribution of zinc(II) coordination numbers is given on Fig. S2; and distribution of various coordination polyhedra for the most widespread coordination numbers 4 - 6 is depicted on Fig. S3. Note, that the number of coordination polyhedra O_4, O_5 and O_6 is substantially non-zero, but these were excluded from analysis as we were interested only in mixed complexes containing L ligands. The number of polyhedra containing three and more nitrogen atoms is also high, but these can appear only if L : Zn^{II} = 2 : 1 and more.

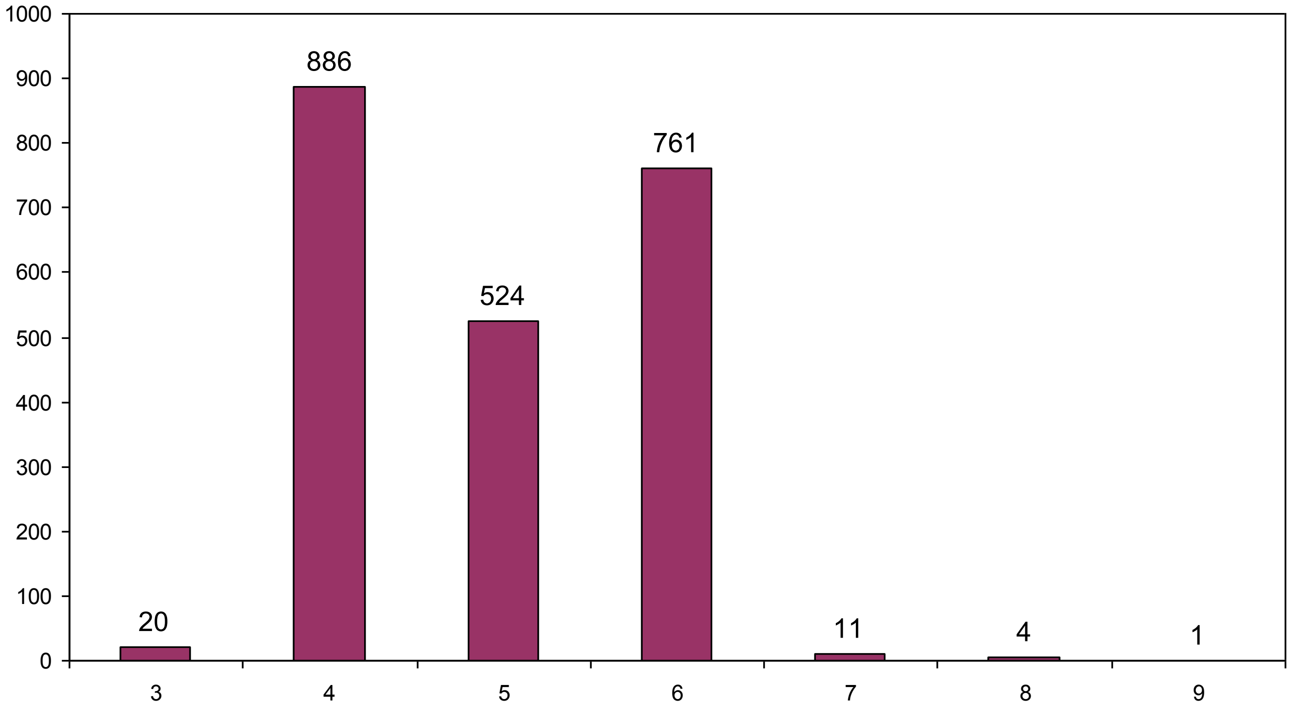

Figure S2 Distribution of coordination numbers for zinc(II) atoms in $Zn^{II}N_xO_y$ coordination polyhedra found in 1473 X-rayed complexes with *bipy*, *bpe* or *bpa* ligands.

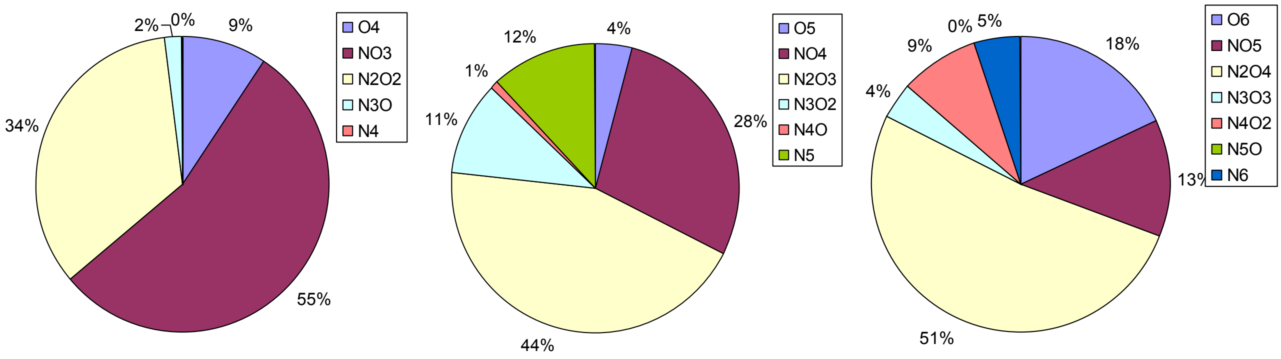

Figure S3 Distribution of various coordination polyhedra for CN(Zn) = 4 (left), 5 (middle) and 6 (right).

Among all zinc(II) complexes with *bipy* analogs, 220 compounds containing 298 symmetrically independent *bpe* ligands (as the target photo-sensitive ligand) were taken to determine possible coordination modes of this linker and probable Zn : L ratios. *bpe* acts as an uncoordinated molecule, terminal ligand or linker in 15, 22 and

261 cases; for *bpa* corresponding values are equal to 4, 9 and 192. The L : Zn^{II} ratio is equal to 0.5, 1 or 2 in 29, 49 and 8 % of complexes. The other values do not exceed 3%.

Table S3 Distribution of L : Zn ratio in previously reported bpe and bpa complexes**.**

L : Zn	0.25	0.33	0.50	0,67	0.75	1.00	1.33	1.50	1.67	2.00	2.50	3.00
N	8	11	118	7	4	199	1	10	1	31	1	14
%	0.02	0.03	**0.29**	0.02	0.01	**0.49**	0.00	0.02	0.00	**0.08**	0.00	0.03

S1.3. Synthetic procedures

General Details: Commercially available reagents were used as received, in particular, $Zn(OAc)_2·2H_2O$ («Roth», Germany, 99%), $Zn(NO_3)_2·6H_2O$ (Chimmed, Russia, «pure»), H_2Me_2Mal («Sigma Aldrich», Switzerland, 98%), H_2Et_2Mal («Sigma Aldrich», Switzerland, 98%), 4,4'-bipyridine («Alfa Aesar», Germany, 98%), 1,2-bis(4-pyridyl)ethane («Sigma Aldrich», Germany, 99%), 1,2-bis(4-pyridyl)ethylene («Sigma Aldrich», Germany, 97%). IR spectra were measured by using a Perkin–Elmer Spectrum 65 instrument by the attenuated total reflection (ATR) method in the range 4000–400 cm^{-1}. CHN analysis was performed by using an automatic CHNS analyzer EuroEA3000 at the Center of Collective Use of IGIC RAS.

Synthesis of new compounds

$[Zn_2(H_2O\text{-}\kappa O)_2(\mu\text{-}\textit{bipy})(\mu_3\text{-}Me_2mal\text{-}\kappa^2O,O')_2]_n$ (1):

A water solution (4 mL) of $Zn(OAc)_2·2H_2O$ (0.033 g, 0.15 mmol) and dimethylmalonic acid (0.020 g, 0.15 mmol) was placed at the bottom of a test tube. Then, a water interphase (6 mL) was carefully layered. A 4 mL acetonitrile solution of 4,4'-bipyridine (0.047 g, 0.3 mmol) was carefully added on the top. The test tube was covered and allowed to stand at room temperature for a week. The resulting colorless crystals are suitable for X-ray diffraction analysis. Crystals of **1** were filtered, washed by water and dried in air at room temperature. IR-spectrum (ATR method), ν/cm^{-1}: 3183 m, 2995 m, 2944 m, 2225 w, 1606 s, 1562 s, 1535 s, 1491 m, 1469 m, 1431 s, 1410 m, 1330 s, 1218 m, 1204 m, 1181 m, 1158 m, 1079 m, 1049 m, 1011 m, 969 m, 945 m, 872 m, 838 m, 813 s, 801 m, 751 s, 733 m, 688 s, 678 s, 630 s, 594 s, 516 m, 498 s, 461 m, 442 m. Calculated (%) for $Zn_2C_{20}H_{24}N_2O_{10}$: %: C, 41.19; H, 4.15; N, 4.8; found (%): C, 41.07; H, 5.30; N, 4.93. The yield of **1** is 0.019 g (44% counting per Zn).

Complexes **2-9** were obtained by a procedure similar to that used in the synthesis of complex **1**.

$\{[Zn(\mu\text{-}\textit{bpe})(\mu\text{-}Me_2mal)] \cdot H_2O\}_n$ (2):

Amounts of reagents: water solution of (10 mL) $Zn(OAc)_2·2H_2O$ (0.100 g, 0.45 mmol) and dimethylmalonic acid (0.060 g, 0.45 mmol), 5 mL of water interphase, acetonitrile solution (10 mL) of bis(4-pyridyl)ethylene (0.166 g, 0.90 mmol). The colorless crystals were grown after 8 days. IR-spectrum (ATR method), ν/cm^{-1}: 3546 m, 3481 w, 3104 w, 3044 w, 3021 w, 2975 w, 2929 w, 2895 w, 2859 w, 1985 w, 1714 w, 1615 s, 1592 s, 1576 s, 1511 m, 1459 m, 1449 m, 1432 s, 1393 s, 1354 m, 1312 m, 1302 m, 1256 m, 1227 w, 1210 m, 1191 m, 1165 m, 1072 m, 1029 s, 996 w, 974 m, 964 m, 887 m, 841 s, 830 s, 802 m, 777 m, 693 m, 590 m, 572 s, 553 s, 460 s, 422 m, 412 m. Calculated (%) for $ZnC_{17}H_{18}N_2O_5$: C, 51.60; H, 4.58; N, 7.08; found (%): C, 51.64; H, 4.69; N, 7.25. The yield of **2** is 0.158g (88% counting per Zn).

$[Zn(\textit{bpe})(Me_2mal)]_2[Zn_2(tpcb)(Me_2mal)_2] \cdot H_2O$ (2a):

A single crystal or powder pattern of **2** was irradiated over 6 hours with Xe laser (λ = 365 nm; 200 W source used with 40 % of the full intensity; see the next Section for details). Reaction product was characterized with

single-crystal and powder diffractions techniques, and ^{1}H NMR. The conversion of **2** to **2a** is 30% based on XRD and ^{1}H NMR data.

[Zn(μ-*bpe*)(HMe$_2$mal)$_2$]$_n$ (3):

Amounts of reagents: water solution (10 mL) of $Zn(NO_3)_2 \cdot 6H_2O$ (0.100 g, 0.34 mmol) and dimethylmalonic acid (0.044 g, 0.33 mmol), 5 mL of water interphase, acetonitrile solution (10mL) of bis(4-pyridyl)ethylene (0.122 g, 0.67 mmol). The colourless crystals were grown after a two week. IR-spectrum (ATR method), ν/cm^{-1}: 3544 w, 3480 w, 3082 w, 3044 w, 2975 w, 2935 w, 2863 w, 2039 w, 1616 s, 1590 s, 1576 m, 1511 w, 1459 w, 1449 w, 1432 m, 1392 m, 1359 m, 1313 m, 1303 m, 1256 w, 1227 w, 1191 m, 1161 w, 1073 w, 1029 s, 996 w, 974 m, 964 m, 887 m, 573 s, 552 s, 453 m, 444 m, 424 m, 405 m. Calculated (%) for $ZnC_{22}H_{24}N_2O_8$: C, 51.83; H, 4.74; N, 5.49; found (%): C, 52.20; H, 4.86; N, 6.25;. The yield of **3** is 0.123 g (72% counting per Zn).

{[Zn(μ-*bpa*)(μ-Me$_2$mal)] · H$_2$O}$_n$ (4):

Amounts of reagents: water solution (0.5 mL) of $Zn(OAc)_2 \cdot 2H_2O$ (0.033 g, 0.15 mmol) and dimethylmalonic acid (0.020 g, 0.15 mmol), 1.3 mL of water interphase, acetonitrile solution (0.6 mL) of 1,2-bis(4-pyridyl)ethane (0.055 g, 0.30 mmol). The colorless crystals were grown after a week. IR-spectrum (ATR method), ν/cm^{-1}: 3557 w, 3484 w, 2983 w, 2930 w, 2865 w, 2225 w.w, 1619 s, 1576 s, 1595 s, 1510 m, 1460 m, 1450 m, 1432 s, 1388 s, 1348 s, 1306 m, 1246 s, 1228 m, 1191 m, 1168 m, 1074 m, 1032 m, 949 s, 886 m, 834 s, 821 m, 802 m, 777 m, 693 m, 590 m, 559 m, 543 s, 493 m, 438 m, 426 m. Calculated (%) for $ZnC_{17}H_{20}N_2O_5$: C, 51.34; H, 5.07; N, 7.04.; found (%): C, 51.02; H, 4.64; N, 7.08. The yield of **4** is 0.038 g (63 % counting per Zn).

{[Zn(*bipy*)(μ-*bipy*)$_{0.5}$(μ-Et$_2$mal)] · H$_2$O}$_n$ (5)

Amounts of reagents: water solution (10 mL) of $Zn(OAc)_2 \cdot 2H_2O$ (0.027 g, 0.12 mmol) and diethylmalonic acid (0.020 g, 0.12 mmol), 5 mL of water interphase, acetonitrile solution (10mL) of 4,4'-bipyridine (0.039 g, 0.25 mmol). The colourless crystals were grown after a two week. IR-spectrum (ATR method), ν/cm^{-1}: 3513 w, 3393 w, 3081 w, 3047 w, 2968 w, 2935 w, 2879 w, 1588 s, 1532 s, 1492 m, 1459 m, 1443 m, 1420 m, 1409 s, 1385 s, 1301 m, 1271 m, 1220 m, 1181 w, 1166 w, 1143 w, 1081 m, 1070 m, 1046 m, 1022 w, 996 m, 964 w, 939 w, 888 w, 878 w, 855 w, 839 w, 810 s, 767 m, 750 m, 731 m, 701 m, 678 m, 643 m, 626 s, 597 m, 576 m, 539 m, 476 s, 430 m, 415 m, 404 s. Calculated (%) for $ZnC_{22}H_{24}N_3O_5$: C, 55.53; H, 5.08; N, 8.83; found (%): C, 55.52; H, 5.32; N, 9.1. The yield of **5** is 0.023 g (38% counting per Zn).

{[Zn(H$_2$O-κO)$_4$(μ-*bipy*)](HEt$_2$mal)$_2$ · *bipy* · 2H$_2$O}$_n$ (6):

Amounts of reagents: water solution (10 mL) of $Zn(NO_3)_2 \cdot 6H_2O$ (0.037 g, 0.12 mmol) and diethylmalonic acid (0.02 g, 0.12 mmol), 4 mL of water interphase, acetonitrile solution (10 mL) of 4,4'-bipyridine (0.039 g, 0.25 mmol). The colourless crystals were grown after a month. IR-spectrum (ATR method), ν/cm^{-1}: 3542 m, 3465 m, 3162 m, 3097 m, 3063 m, 2970 m, 2932 m, 2878 m, 2343 w, 1963 w, 1698 m, 1671 m, 1607 s, 1596 s, 1536 m, 1490 m, 1453 m, 1408 s, 1378 s, 1307 s, 1227 m, 1182 m, 1133 m, 1104 m, 1072 m, 1039 m, 1008 m, 996 m, 982 m, 930 m, 833 s, 804 s, 764 s, 730 s, 632 s, 616 s, 570 s, 483 s, 450 s, 413 s. Calculated (%) for

$ZnC_{34}H_{50}N_4O_{14}$: C, 50.78; H, 6.27; N, 6.97; found (%): C, 51.02; H, 6.43; N, 7.15. The yield of **6** is 0.037 g (38% counting per Zn).

{[Zn(μ- *bpe*)(μ-Et$_2$mal)]·0.25*bpe*}$_n$ (7):

Amounts of reagents: water solution of (10 mL) $Zn(OAc)_2{\cdot}2H_2O$ (0.100 g, 0.45 mmol) and diethylmalonic acid (0.073 g, 0.45 mmol), 4 mL of water interphase, acetonitrile solution (10 mL) of bis(4-pyridyl)ethylene (0.166 g, 0.90 mmol). The pale-yellow crystals were grown after a week. IR-spectrum (ATR method), ν/cm^{-1}: 3044 w, 3017 w, 2958 w, 2937 w, 2874 w, 1602 s, 1512 m, 1437 m, 1374 m, 1361 m, 1335 m, 1287 m, 1264 m, 1212 m, 1164 m, 1141 w, 1074 m, 1030 m, 991 m, 967 m, 952 m, 880 w, 844 m, 803 m, 768 w, 750 w, 699 m, 599 m, 574 m, 549 s, 492 w, 469 w, 430 m, 417 w, 409 m, 404 w. Calculated (%) for $ZnC_{22}H_{22.5}N_{2.5}O_4$: C, 58.55; H, 5.02; N,7.76; found (%): C, 58.37; H, 4.91 ; N, 7.51. The yield of **7** is 0.168 g (82% counting per Zn).

[Zn(μ-*bpe*)$_{0.75}$(μ-tpcb)$_{0.25}$(μ-Et$_2$mal)]$_n$ (7a):

A single crystal or powder pattern of **7** was irradiated over 6 hours with Xe laser (λ = 365 nm; 200 W source used with 40 % of the full intensity; see the next Section for details). Reaction product was characterized with single-crystal and powder diffractions techniques, and ^{1}H NMR. The conversion of **7** to **7a** is 100% based on XRD and ^{1}H NMR data.

[Zn(H$_2$O-κO)$_4$(*bpe*)$_2$](HEt$_2$mal)$_2$ (8)

Amounts of reagents: water solution (10 mL) of $Zn(NO_3)_2{\cdot}6H_2O$ (0.100 g, 0.33 mmol) and diethylmalonic acid (0.054 g, 0.33 mmol), 5 mL of water interphase, acetonitrile solution (10 mL) of bis(4-pyridyl)ethylene (0.123 g, 0.67 mmol). The pale-yellow crystals were grown after a week. IR-spectrum (ATR method), ν/cm^{-1}: 3040 w, 2957 w, 2937 w, 2872 w, 1602 s, 1512 m, 1438 m, 1375 m, 1361 m, 1335 m, 1287 m, 1263 m, 1212 m, 1164 m, 1141 m, 1074 m, 1030 s, 992 m, 966 m, 951 m, 879 w, 844 s, 802 m, 767 w, 750 w, 699 m, 598 m, 574 m, 549 s, 495 m, 429 m, 420 m, 403 m. Calculated (%) for $ZnC_{38}H_{48}N_4O_{12}$: C, 55.78; H, 5.91; N, 6.85; found (%): C, 55.57; H, 5.64; N, 6.72. The yield of **8** is 0.119 g (43 % counting per Zn).

[Zn(H$_2$O-κO)$_4$(*bpe*)$_2$]$_{0.15}$[Zn(H$_2$O)$_4$(tpcb)]$_{0.85}$(HEt$_2$mal)$_4$ (8a):

A single crystal or powder pattern of **8** was irradiated over 6 hours with Xe laser (λ = 365 nm; 200 W source used with 40 % of the full intensity; see the next Section for details). Reaction product was characterized with single-crystal and powder diffractions techniques, and ^{1}H NMR. The conversion of **8** to **8a** is 90% based on XRD and ^{1}H NMR data.

{[Zn(μ-*bpa*)(μ-Et$_2$mal)] · 0.38H$_2$O}$_n$ (9):

Amounts of reagents: water solution of (10 mL) $Zn(OAc)_2{\cdot}2H_2O$ (0.100 g, 0.45 mmol) and diethylmalonic acid (0.073 g, 0.45 mmol), 2 mL of water interphase, acetonitrile solution (5 mL) of 1,2-bis(4-pyridyl)ethane (0.168 g, 0.9 mmol). The colorless crystals were grown after 8 days. IR-spectrum (ATR method), ν/cm^{-1}: 3066 w, 3043 w, 2957 m, 2933 w, 2874 w, 1603 s, 1506 m, 1456 m, 1432 m, 1373 s, 1337 m, 1289 m, 1266 m, 1225 m, 1211 m, 1163 m, 1137 w, 1072 m, 1032 s, 954 w, 883 w, 837 s, 804 s, 703 m, 597 m, 545 s, 499 m, 424 m, 417

m, 407 m. Calculated (%) for $ZnC_{19}H_{22.75}N_2O_{4.38}$: C, 55.04; H, 5.53; N, 6.76; found (%): C, 55.37; H, 5.38; N, 6.86. The yield of **9** is 0.110 g (58% counting per Zn).

S1.4. Crystallography

Single crystals of **1-9** were obtained from reaction mixtures. The intensities of reflections were measured with a Bruker Apex II DUO CCD diffractometer using graphite monochromated MoK$_{\alpha}$ radiation (λ = 0.71073 Å) at 120.0(2) K. Intensity data for **2a** were collected at the at the K4.4 station of the Kurchatov Center for Synchrotron Radiation and Nanotechnology in Moscow (Russia) at a wavelength of 0.9699 Å using a MAR CCD 165 detector and merged using SCALA.[S4] Data collection was performed at low temperature [100 K] using an Oxford CryoJet from Oxford Cryosystems Ltd. The structures were solved by the direct method and refined by full-matrix least squares against F^2. Non-hydrogen atoms were refined anisotropically except some disordered atoms. The disordered fragments, particularly, one carbon atom of *bipy* in **1**, solvent *bpe* molecule in **7**, one ethyl fragment in **9**, a methyl group and all carbon atoms of *bpe* and tpcb ligands in **2a** were refined isotropically. A number of EADP, ISOR, SADI, RIGU and DFIX instructions were applied to refine some moieties, especially, in crystals of **2a**, **7**, **8a** disordered by symmetry or containing disordered fragments. TWIN/BASF refinement was performed for **9**. Positions of hydrogen atoms were calculated and all were included in the refinement by the riding model with U_{iso}(H) = 1.5U_{eq}(X) for methyl groups and water molecules, and U_{iso}(H) = 1.2U_{eq}(X) for other atoms. All calculations were made using the SHELXL2014[S5] and OLEX2[S6] program packages. Experimental details and crystal parameters are listed in Tables S4 and S5.

Then, single crystals **2, 7** and **8** containing closely packed *bpe* ligands were irradiated over 6 hours with Xe laser (λ = 365 nm; 200 W source used with 40 % of the full intensity) on air. XRD confirmed that these compounds underwent single-crystal-to-single-crystal photoreactions to afford, respectively, **2a, 7a** and **8a**, accompanied with loss of some uncoordinated water by **2a**. Careful inspection of occupancies of carbon atoms of ethylene or cyclobutane fragments indicated that only **7a** underwent 100% conversion. **8a** contained 15 % of initial substance, and **2a** contained 50% of **2**.

Table S4 Crystallographic Data and Refinement Parameters for Zinc(II) Dimethylmalonates

Compound	$[Zn_2(H_2O)_2$(*bipy*)$(Me_2$mal$)_2]$ (**1**)	[Zn(*bpe*)$(Me_2$mal)] · H_2O (**2**)	[Zn(*bpe*)$(Me_2$mal)$]_2[Zn_2$(tpcb)$(Me_2$mal$)_2]$ · H_2O (**2a**)	[Zn(*bpe*)$(HMe_2$mal$)_2]$ (**3**)	[Zn(*bpa*)$(Me_2$mal)] · H_2O (**4**)
CCDC	1568619	1568620	1568621	1568622	1568623
Formula	$C_{20}H_{24}N_2O_{10}Zn_2$	$C_{17}H_{18}N_2O_5Zn$	$C_{17}H_{17}N_2O_{4.25}Zn$	$C_{22}H_{24}N_2O_8Zn$	$C_{17}H_{20}N_2O_5Zn$
Fw	583.15	395.70	382.69	509.80	397.72
Crystal System	Orthorhombic	Monoclinic	Orthorhombic	Monoclinic	Monoclinic
Space group	*P n n m*	$P\ 2_1/c$	*P n n a*	*C 2/c*	$P\ 2_1/c$
Wavelength (Å)	0.71073	0.71073	0.9699	0.71073	0.71073
a (Å)	7.4159(16)	8.2852(11)	8.3000(17)	18.0211(8)	8.4434(14)
b (Å)	19.318(4)	10.4999(14)	10.400(2)	5.8795(2)	10.3811(18)
c (Å)	7.3457(16)	21.684(3)	19.740(4)	21.7192(12)	21.551(4)
β (°)	90	117.282(3)	90	113.890(1)	115.928(4)
V (Å^3)	1052.3(4)	1676.5(4)	1704.0(6)	2104.10(17)	1698.8(5)
Z	2	4	4	4	4
d_c (g/cm^3)	1.840	1.568	1.492	1.609	1.555
μ (mm^{-1})	2.343	1.496	3.318	1.222	1.476
F(000)	596	816	788	1056	824
I_{hkl} coll/uniq	15845 / 2674	19935 / 5502	10296 / 1696	6512 / 2296	25908 / 7954
R_{int}	0.094	0.061	0.087	0.044	0.053
Obs.refl. / N	1859 / 103	4217 / 236	976 / 117	2562 / 150	6155 / 232
R,[a] % [I > 2	0.066	0.051	0.093	0.026	0.070
R_w,[b] %	0.145	0.101	0.214	0.069	0.153
GOF[c]	1.04	1.00	1.07	1.13	1.00

[a] $R = \Sigma||F_o|-|F_c||/\Sigma|F_o|$. [b] $R_w = [\Sigma(w(F_o^2-F_c^2)^2)/\Sigma(w(F_o^2))]^{1/2}$. [c] $GOF = [\Sigma w(F_o^2-F_c^2)^2/(N_{obs}-N_{param})]^{1/2}$

Table S5 Crystallographic data and refinement parameters for zinc(II) diethylmalonates

Compound	$[Zn(\mathit{bipy})_{1.5}(Et_2mal)] \cdot H_2O$ (**5**)	$[Zn(H_2O)_4(\mathit{bipy})] \cdot 2HEt_2mal \cdot \mathit{bipy} \cdot 2H_2O$ (**6**)	$[Zn(\mathit{bpe})(Et_2mal)] \cdot 0.25\mathit{bpe}$ (**7**)	$[Zn(\mathit{bpe})_{0.75}(tpcb)_{0.25}(Et_2mal)]$ (**7a**)	$[Zn(H_2O)_4(\mathit{bpe})_2](HEt_2mal)_2$ (**8**)	$[Zn(H_2O)_4(\mathit{bpe})_2]_{0.15}[Zn(H_2O)_4(tpcb)]_{0.85}(HEt_2mal)_4$ (**8a**)	$[Zn(\mathit{bpa})(Et_2mal)] \cdot 0.38H_2O$ (**9**)
CCDC	1568624	1568625	1568626	1568627	1568628	1568629	1568630
Formula	$C_{22}H_{24}N_3O_5Zn$	$C_{34}H_{50}N_4O_{14}Zn$	$C_{22}H_{22.5}N_{2.5}O_4Zn$	$C_{22}H_{22.5}N_{2.5}O_4Zn$	$C_{38}H_{48}N_4O_{12}Zn$	$C_{38}H_{48}N_4O_{12}Zn$	$C_{19}H_{22.75}N_2O_{4.38}Zn$
Fw	475.81	804.15	451.29	451.29	818.17	818.17	414.51
Crystal System	Monoclinic	Monoclinic	Orthorhombic	Orthorhombic	Monoclinic	Monoclinic	Orthorhombic
Space group	*P 2₁/c* → $P\ 2_1/c$	$C\ 2/c$	$C\ m\ c\ m$	$P\ b\ c\ n$	$C\ 2/m$	$C\ 2/c$	$C\ 2\ 2\ 2_1$
Wavelength (Å)	0.71073	0.71073	0.71073	0.71073	0.71073	0.71073	0.71073
a (Å)	11.1832(8)	22.6008(12)	9.8266(5)	9.845(8)	22.881(2)	26.8028(19)	24.662(3)
b (Å)	8.2422(6)	11.4154(6)	22.0654(12)	22.082(17)	6.8976(6)	6.8933(5)	37.180(5)
c (Å)	23.8320(17)	17.1552(9)	22.1641(11)	21.905(17)	14.1573(12)	23.0690(17)	8.7826(12)
β (°)	99.143(2)	120.308(1)	90	90	117.532(2)	110.021(1)	90
V (Å^3)	2168.8(3)	3821.1(4)	4805.8(4)	4762(6)	1981.3(3)	4004.6(5)	8053.0(19)
Z	4	4	8	8	2	4	16
d_c (g/cm^3)	1.457	1.398	1.247	1.259	1.371	1.357	1.368
μ (mm^{-1})	1.171	0.713	1.050	1.059	0.686	0.679	1.247
F(000)	988	1696	1872	1872	860	1720	3452
I_{hkl} coll/uniq	23649 / 6785	27960 / 7041	22224 / 3498	40353 / 7285	17312 / 4490	25740 / 6154	41027 / 12430
R_{int}	0.073	0.029	0.082	0.202	0.039	0.050	0.103
Obs.refl. / N	4755 / 282	5947 / 264	2496 / 209	3089 / 252	3929 / 182	3847 / 314	7821 / 461
R,[a] % [I > 2σ(I	0.047	0.029	0.052	0.114	0.052	0.055	0.065
R_w,[b] %	0.103	0.075	0.140	0.237	0.132	0.176	0.150
GOF[c]	1.01	1.02	1.01	1.01	1.18	1.03	1.02
Flack	-	-	-	-	-	-	0.01(2)

[a] $R = \Sigma||F_o| - |F_c||/\Sigma|F_o|$. [b] $R_w = [\Sigma(w(F_o^2 - F_c^2)^2)/\Sigma(w(F_o^2))]^{1/2}$. [c] $GOF = [\Sigma w(F_o^2 - F_c^2)^2/(N_{obs} - N_{param})]^{1/2}$

S1.5. Powder X-Ray diffraction.

Phase composition of the bulk samples was confirmed with powder XRD. Powder patterns were measured on a Bruker D8 Advance diffractometer at room temperature with LynxEye detector and Ge(111) monochromator, $\lambda(CuK\alpha_1) = 1.54060$ Å, θ/2θ scan from 4° to 60°. The powder patterns were modeled with the Rietveld method using Bruker TOPAS5[S7] software. Fundamental parameters approach(Cheary & Coelho, 1992) was used for profile fitting. The preferred orientation was taken into account with the spherical harmonics approach(Järvinen, 1993).

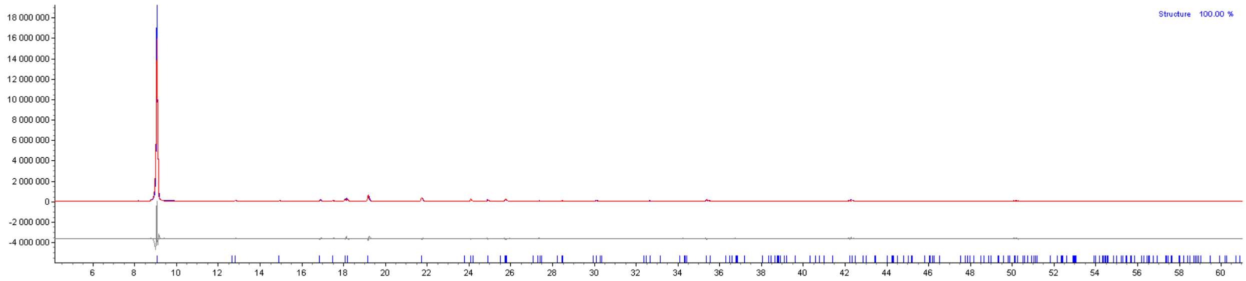

Figure S4 The experimental (blue) and calculated (red) powder patterns for $[Zn_2(H_2O)_2(\mathit{bipy})(Me_2mal)_2]$ (**1**) and their difference (grey). Smooth residual curve indicates purity of the sample; high R_{wp}/R_{bragg} = 20.28/4.95 % values are related to prominent preferred orientation.

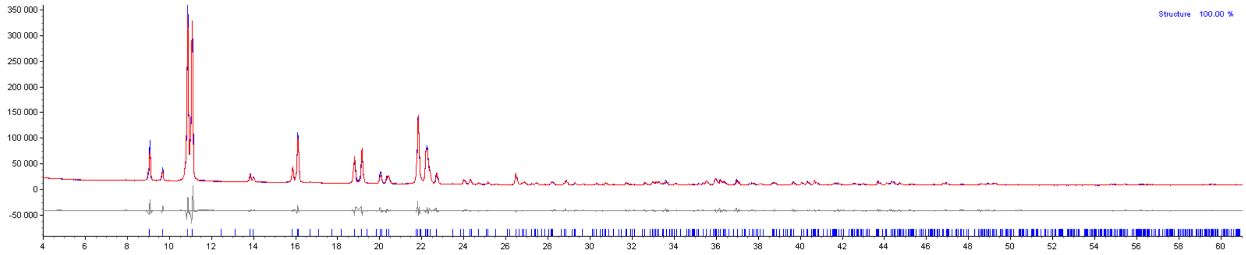

Figure S5 The experimental (blue) and calculated (red) powder patterns for $[Zn(\mathit{bpe})(Me_2mal)] \cdot H_2O$ (**2**) and their difference (grey). R_{wp}/R_{bragg} = 6.04/1.42 % indicate purity of the sample.

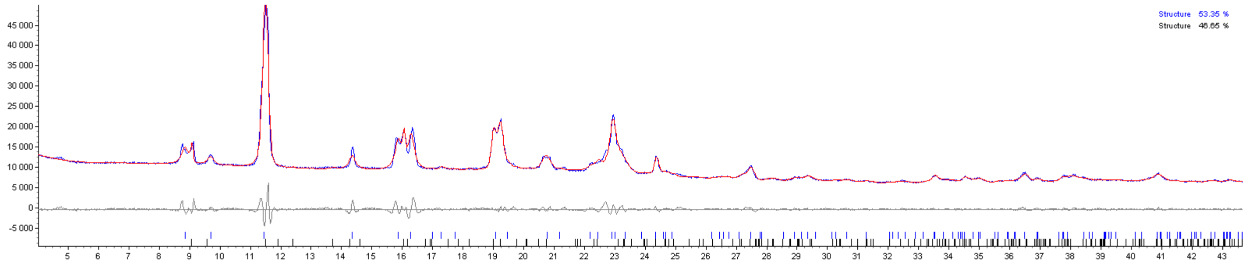

Figure S6 The experimental (blue) and calculated (red) powder patterns for $[Zn(\mathit{bpe})(Me_2mal)] \cdot H_2O$ (**2**) irradiated for 6 hours. Rietveld analysis indicates that the sample contains the $[Zn(\mathit{bpe})(Me_2mal)] \cdot H_2O$ and $[Zn(\mathit{bpe})(Me_2mal)]_2[Zn_2(tpcb)(Me_2mal)_2] \cdot H_2O$ phases in 1 : 1 ratio. R_{wp} = 3.479% and R_{bragg} = 0.387/0.481%. The blue line is the experimental pattern, the fuchsia line is the calculated pattern, and the grey line is the difference curve.

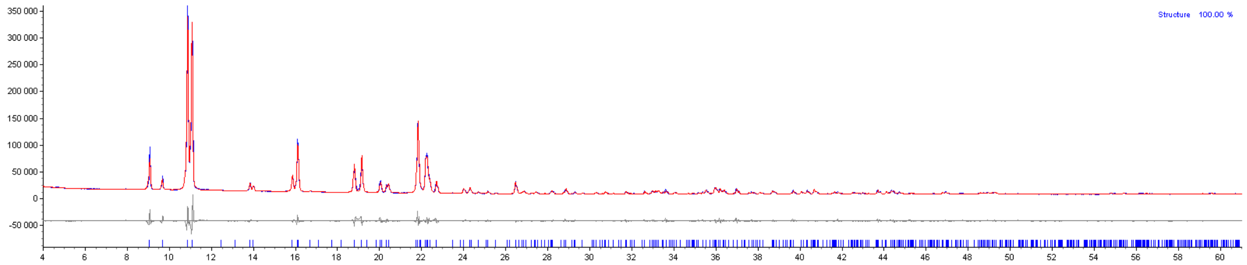

Figure S7 XRD pattern for [Zn(μ-*bpa*)(μ-Me_2mal)] · H_2O}$_n$ (**4**). Rietveld analysis indicates purity of the sample. R_{wp}/R_{bragg} = 6.195/1.106 %. The blue line is the experimental pattern, the fuchsia line is the calculated pattern, and the grey line is the difference curve.

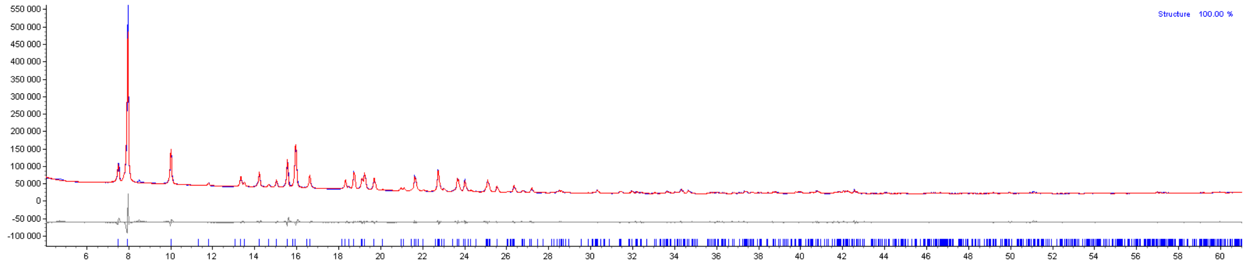

Figure S8 The experimental (blue) and calculated (red) powder patterns for [Zn(*bipy*)$_{1.5}$(Et_2mal)] · H_2O (**5**) and their difference (grey). R_{wp}/R_{bragg} = 3.32/0.67 % indicate purity of the sample.

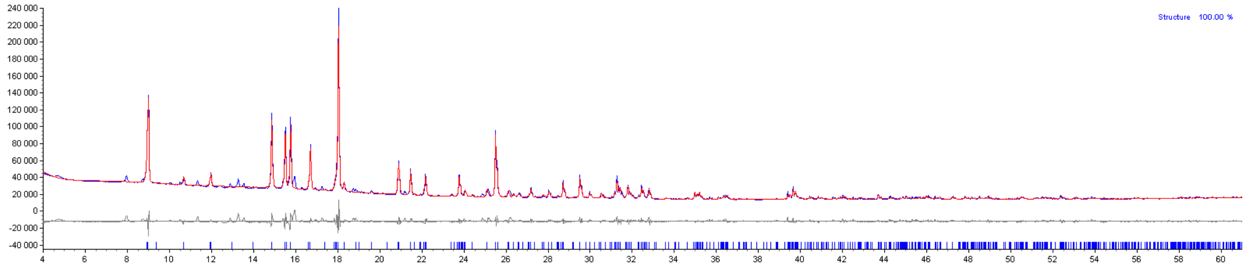

Figure S9 The experimental (blue) and calculated (red) powder patterns for [Zn(H_2O)$_4$(*bipy*)](HEt_2mal)$_2$ · *bipy* · 2H_2O (**6**) and their difference (grey). Although some impurity is present in the sample, R_{wp}/R_{bragg} = 4.98/1.16 % indicate that the sample consists mainly of the target product.

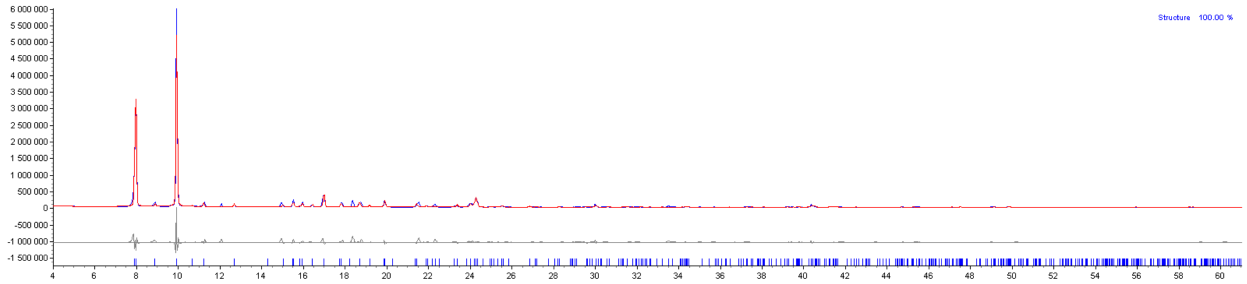

Figure S10 The experimental (blue) and calculated (red) powder patterns for [Zn(*bpe*)(Et_2mal)] (**7**) and their difference (grey). Smooth residual curve and R_{wp}/R_{bragg} = 17.73/3.88 % indicate that the

sample exhibit strong prefered orientation and consists mainly from the target substance. Some impurity is present that we failed to determine.

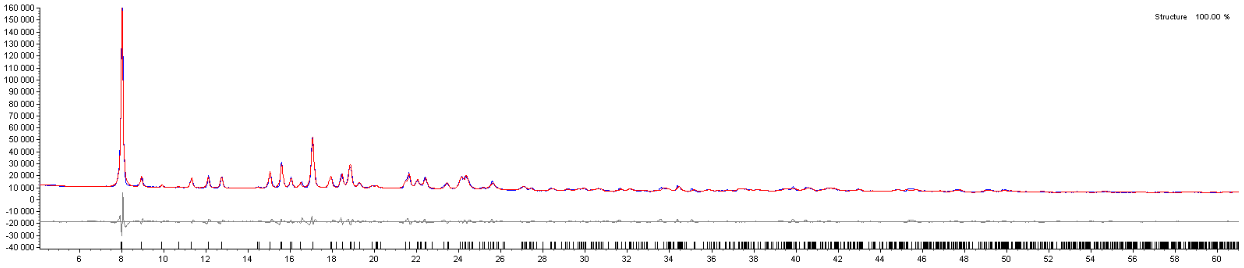

Figure S11 The experimental (blue) and calculated (red) powder patterns for [Zn(*bpe*)(Et_2mal)] (**7**) irradiated for 6 hours. Rietveld analysis indicates purity of the sample. R_{wp} = 5.296% and R_{bragg} = 1.030%. The blue line is the experimental pattern, the fuchsia line is the calculated pattern, and the grey line is the difference curve.

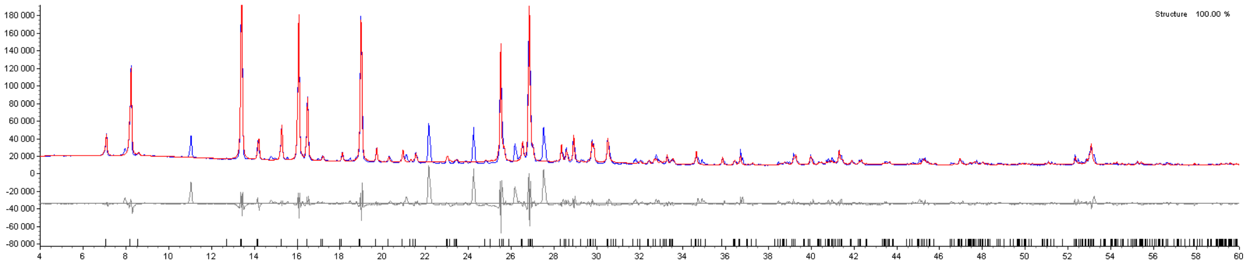

Figure S12 The experimental (blue) and calculated (red) powder patterns for [Zn(H_2O)$_4$(*bpe*)$_2$](HEt_2mal)$_2$ (**8**) and their difference (grey). Rietveld analysis indicates that the sample consists mainly from the target substance. Some impurity is present that we failed to determine.

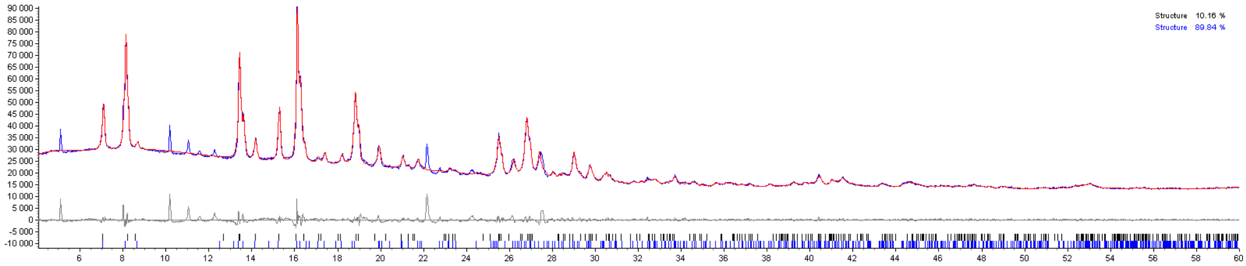

Figure S13 The experimental (blue) and calculated (red) powder patterns for [Zn(H_2O)$_4$(*bpe*)$_2$](HEt_2mal)$_2$ (**8**) irradiated for 6 hours. Rietveld analysis indicates that the sample consists of [Zn(H_2O)$_4$(*bpe*)$_2$](HEt_2mal)$_2$ and [Zn(H_2O)$_4$(*bpe*)$_2$]$_{0.15}$[Zn(H_2O)$_4$(tpcb)]$_{0.85}$(HEt_2mal)$_4$ in 1 : 9 ratio and contains some impurity. Rwp = 3.193% and Rbragg = 0.215/0.304%. The blue line is the experimental pattern, the fuchsia line is the calculated pattern, and the grey line is the difference curve.

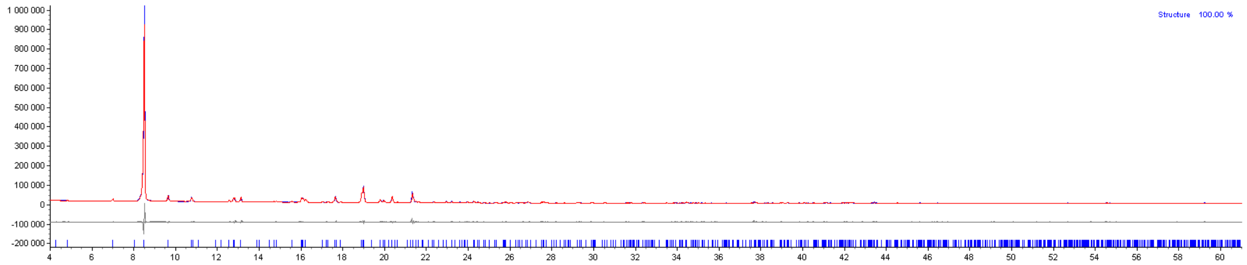

Figure S14 The experimental (blue) and calculated (red) powder patterns for [Zn(*bpa*)(Et_2mal)] · 0.38H_2O (**9**) and their difference (grey). Although some impurity is present in the sample, R_{wp}/R_{bragg} = 6.31/1.00 % indicate purity of the sample.

S1.6. ^{1}H NMR Analysis of acidified 2a, 7a and 8a in d_6-DMSO

1H NMR spectra were recorded on a 300 MHz Bruker FT-NMR spectrometer with TMS as an internal reference. As even moderate heating of d_6-DMSO solutions of reaction products **2a**, **7a** and **8a** causes cleavage of tpcb, the samples were dissolved using a drop of HNO_3 at room temperature.

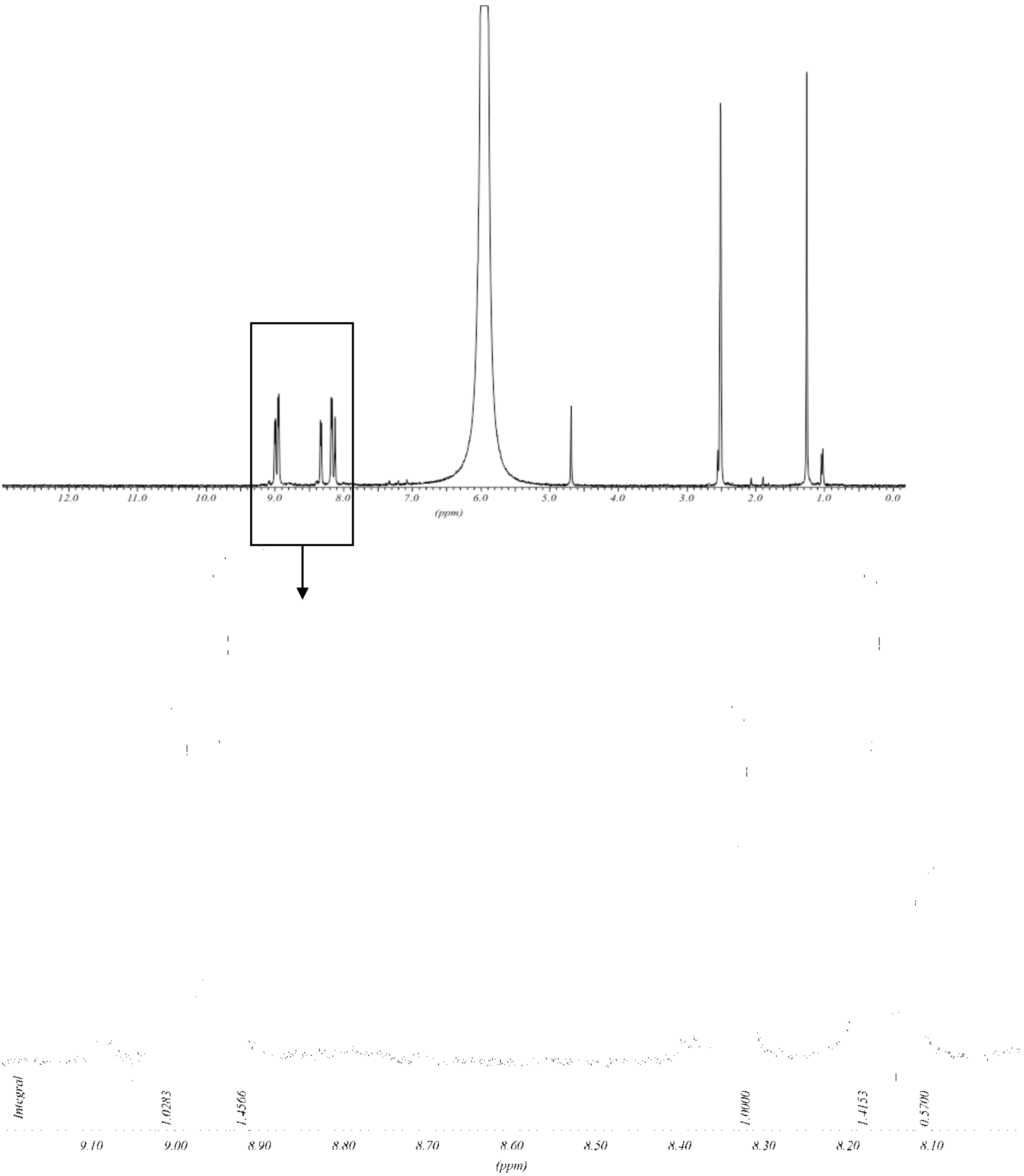

Figure S15 ^{1}H NMR spectrum of **2a** dissolved in d_6-DMSO using a drop of HNO_3.

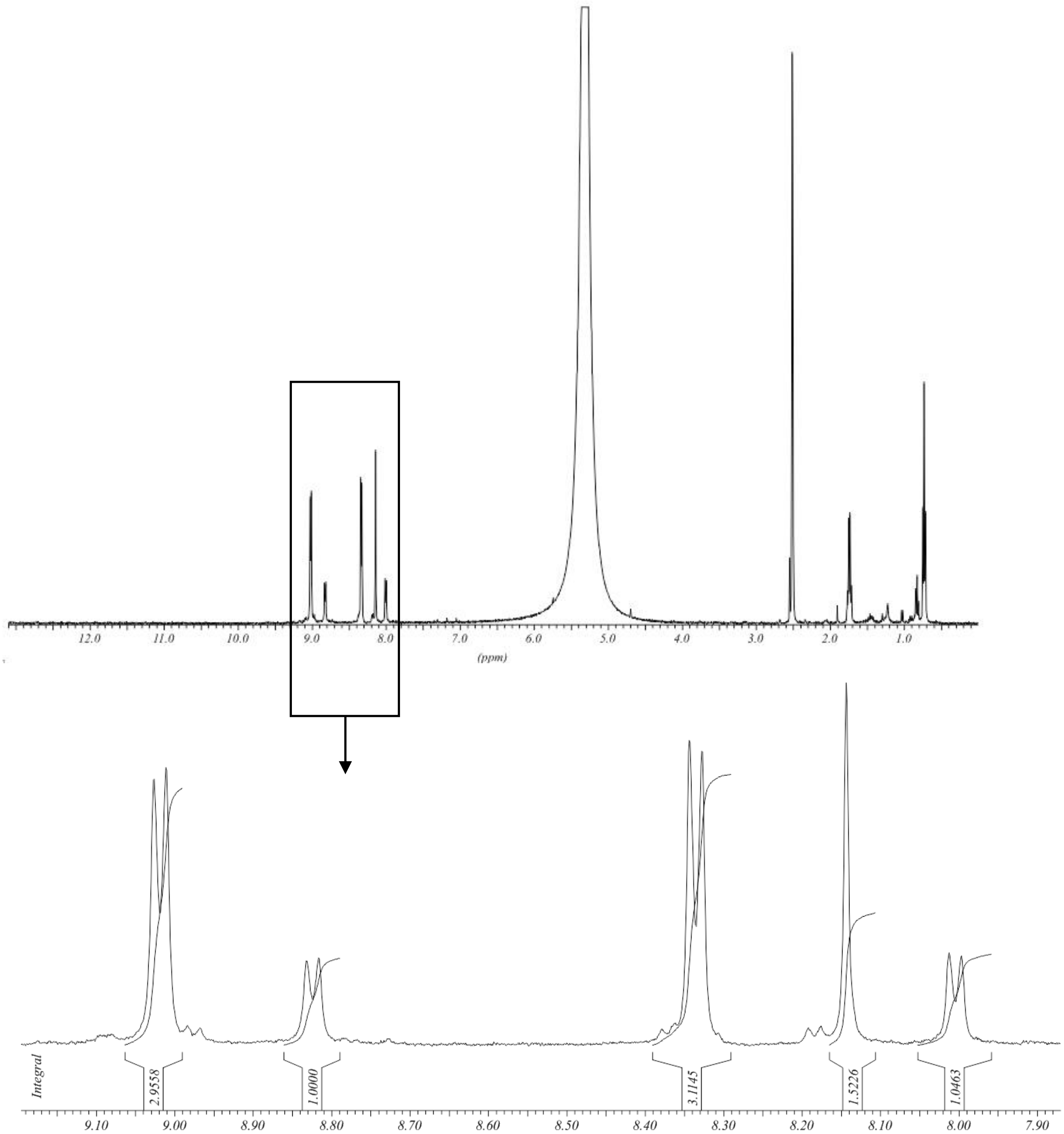

Figure S16 ^{1}H NMR spectrum of **7a** dissolved in d_6-DMSO using a drop of HNO_3.

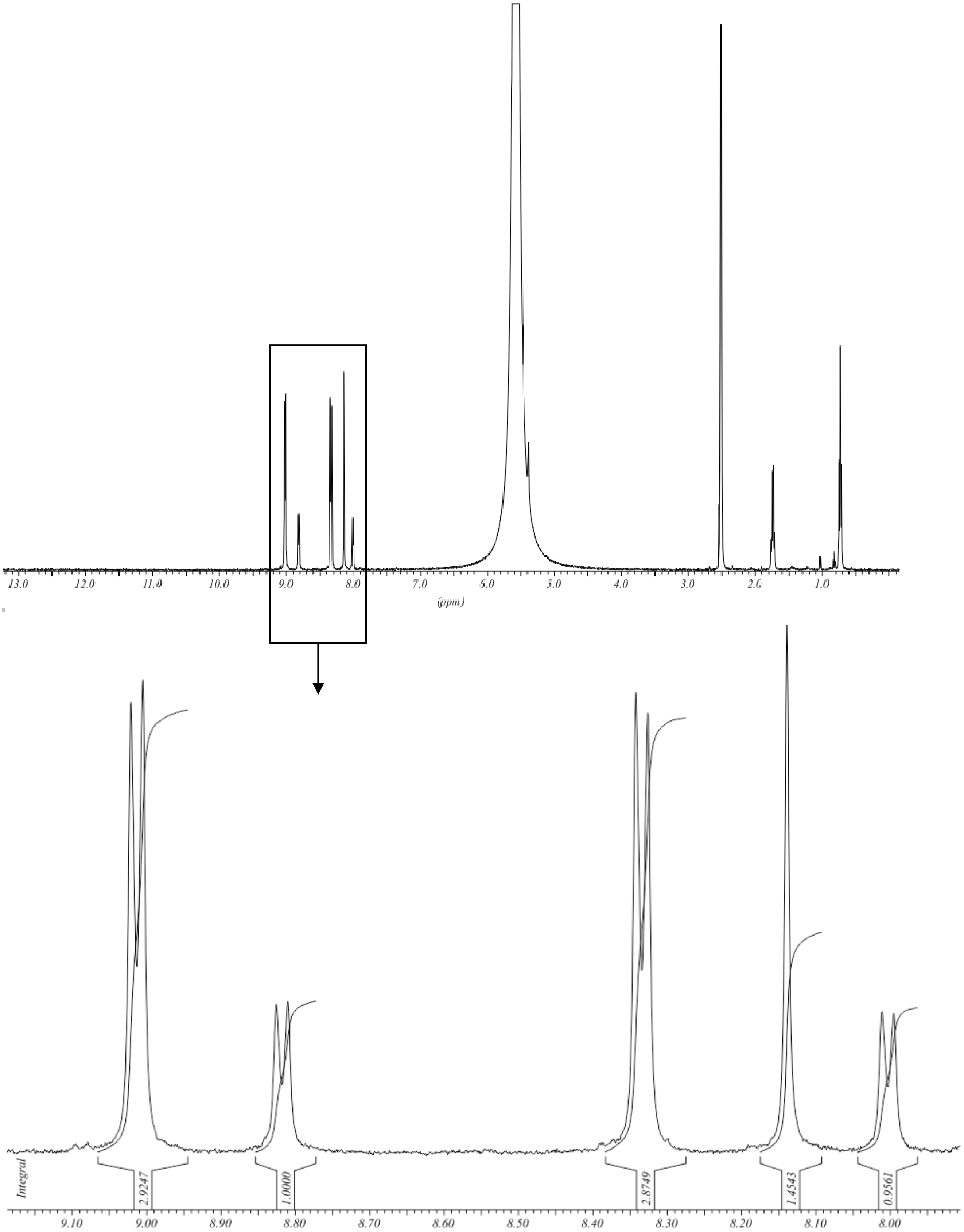

Figure S17 ^{1}H NMR spectrum of **8a** dissolved in d_6-DMSO using a drop of HNO_3.

S2. Systre INPUT files for all 2D and 3D coordination polymers

1

```
crystal
name 0.5(C40 H48 N4 O20 Zn4)
cell 7.4159 19.3180 7.3457 90.000 90.000 90.000
group Pnnm
atom 1 3 0.75138 0.81710 0.50000
edge 1 0.4886 0.7189 0.5000
edge 1 0.9886 0.7811 1.0000
edge 1 0.9886 0.7811 0.0000
atom 2 4 0.48860 0.71895 0.50000
edge 2 0.7514 0.8171 0.5000
edge 2 0.2514 0.6829 0.0000
edge 2 0.2514 0.6829 1.0000
edge 2 1.5114 0.2811 0.5000
end
```

2

```
crystal
name C17 H16 N2 O4 Zn, H2 O
cell 8.2852 10.4999 21.6840 90.000 117.280 90.000
group P121/c1
atom 1 4 0.05990 0.49185 0.31425
edge 1 -0.0599 0.9918 0.1858
edge 1 -0.0599 -0.0082 0.1858
edge 1 0.9401 1.5082 0.6858
edge 1 -0.0599 -0.4918 0.6858
end
```

2a - novel topology

```
Coordination sequences
----------------------
Ti1:  1  2  3  4   5   6   7   8    9   10
Num   4 11 24 49  82 128 180 235  302  377
Cum   5 16 40 89 171 299 479 714 1016 1393
----------------------
Zn1:  1  2  3  4   5   6   7   8    9   10
Num   4 12 28 50  84 130 180 240  302  372
Cum   5 17 45 95 179 309 489 729 1031 1403
----------------------
Zn2:  1  2  3  4   5   6   7   8   9   10
Num   4  9 20 43  78 122 172 227 292  369
Cum   5 14 34 77 155 277 449 676 968 1337
----------------------
TD10=1377

Vertex symbols for selected sublattice
--------------------------------------
Ti1 Point symbol:{4.6^4.8}
```

Extended point symbol:[4.8(5).6.6.6.6]

Zn1 Point symbol:{6^5.8}
Extended point symbol:[6.6.6.6.6(2).8(2)]

Zn2 Point symbol:{4^3.6^3}
Extended point symbol:[4.4.4.6(2).6.6]

Point symbol for net: {4.6^4.8}{4^3.6^3}{6^5.8}
4,4,4-c net with stoichiometry (4-c)(4-c)(4-c); 3-nodal net

____

crystal
name C17 H16 N2 O4 Zn, H2 O / B2 to K4
cell 8.2852 10.4999 21.6840 90.000 117.280 90.000
group P21
atom 1 4 0.75000 0.50000 0.75000
edge 1 0.9401 0.9919 0.9358
edge 1 0.9401 -0.0081 0.9358
edge 1 0.0599 1.0082 0.5642
edge 1 1.0599 0.0082 0.5642
atom 2 4 0.05990 0.00815 0.56425
edge 2 -0.0599 -0.4918 0.4358
edge 2 -0.0599 0.5082 0.4358
edge 2 0.7500 -0.5000 0.7500
edge 2 -0.2500 0.5000 0.7500
atom 3 4 0.94010 0.99185 0.93575
edge 3 1.0599 1.4919 1.0642
edge 3 1.0599 0.4919 1.0642
edge 3 0.7500 0.5000 0.7500
edge 3 0.7500 1.5000 0.7500
end

4
crystal
name C17 H18 N2 O4 Zn, H2 O
cell 8.4434 10.3811 21.5510 90.000 115.930 90.000
group P121/c1
atom 1 4 0.93529 0.99201 0.18412
edge 1 1.0647 0.4920 0.3159
edge 1 1.0647 1.4920 0.3159
edge 1 1.0647 0.0080 -0.1841
edge 1 0.0647 2.0080 -0.1841
end

____

5
crystal
name C22 H22 N3 O4 Zn, H2 O
cell 11.1832 8.2422 23.8320 90.000 99.140 90.000
group P121/c1

```
atom 1 3 0.55981 0.07828 0.32295
edge 1 0.4402 0.5783 0.1770
edge 1 0.4402 -0.4217 0.1770
edge 1 0.4402 0.9217 0.6770
end
```

---

7 - novel topology
Structure consists of 3D framework with Zn

Coordination sequences

```
----------------------
Zn1:  1  2  3   4   5   6   7    8    9   10
Num   4 12 30  70 130 212 315  430  545  682
Cum   5 17 47 117 247 459 774 1204 1749 2431
----------------------
Zn2:  1  2  3   4   5   6   7    8    9   10
Num   4 12 30  70 130 212 315  430  545  682
Cum   5 17 47 117 247 459 774 1204 1749 2431
----------------------
Zn3:  1  2  3   4   5   6   7    8    9   10
Num   4 12 30  70 130 212 315  430  545  682
Cum   5 17 47 117 247 459 774 1204 1749 2431
----------------------
Zn4:  1  2  3   4   5   6   7    8    9   10
Num   4 12 30  70 130 212 315  430  545  682
Cum   5 17 47 117 247 459 774 1204 1749 2431
----------------------
Zn5:  1  2  3   4   5   6   7    8    9   10
Num   4 12 30  70 130 212 315  430  545  682
Cum   5 17 47 117 247 459 774 1204 1749 2431
----------------------
Zn6:  1  2  3   4   5   6   7    8    9   10
Num   4 12 30  70 130 212 315  430  545  682
Cum   5 17 47 117 247 459 774 1204 1749 2431
----------------------
Zn7:  1  2  3   4   5   6   7    8    9   10
Num   4 12 30  70 130 212 315  430  545  682
Cum   5 17 47 117 247 459 774 1204 1749 2431
----------------------
Zn8:  1  2  3   4   5   6   7    8    9   10
Num   4 12 30  70 130 212 315  430  545  682
Cum   5 17 47 117 247 459 774 1204 1749 2431
----------------------
TD10=2431
```

Vertex symbols for selected sublattice

```
--------------------------------------
Zn1 Point symbol:{6^5.8}
Extended point symbol:[6.6.6.6.6(2).8(2)]
--------------------------------------
```

Zn2 Point symbol:{6^5.8}
Extended point symbol:[6.6.6.6.6(2).8(2)]

Zn3 Point symbol:{6^5.8}
Extended point symbol:[6.6.6.6.6(2).8(2)]

Zn4 Point symbol:{6^5.8}
Extended point symbol:[6.6.6.6.6(2).8(2)]

Zn5 Point symbol:{6^5.8}
Extended point symbol:[6.6.6.6.6(2).8(2)]

```
crystal
name C19 H20 N2 O4 Zn
cell 22.1641 9.8266 22.0654 90.000 90.000 90.000
group P1
atom 1 4 0.45563 0.00000 0.20098
edge 1 0.5444 -0.5000 0.2990
edge 1 0.5444 0.5000 0.2990
edge 1 0.5444 -1.0000 -0.2010
edge 1 0.0444 1.0000 0.2010
atom 2 4 0.95563 0.00000 0.79902
edge 2 1.0444 -0.5000 0.7010
edge 2 1.0444 0.5000 0.7010
edge 2 1.0444 -1.0000 1.2010
edge 2 0.5444 1.0000 0.7990
atom 3 4 0.04437 0.50000 0.70098
edge 3 -0.0444 0.0000 0.7990
edge 3 -0.0444 1.0000 0.7990
edge 3 -0.0444 -0.5000 0.2990
edge 3 0.4556 1.5000 0.7010
atom 4 4 0.54437 0.50000 0.29902
edge 4 0.4556 0.0000 0.2010
edge 4 0.4556 1.0000 0.2010
edge 4 0.4556 -0.5000 0.7010
edge 4 0.9556 1.5000 0.2990
atom 5 4 0.45563 0.50000 0.70098
edge 5 0.5444 1.0000 0.7990
edge 5 0.5444 0.0000 0.7990
edge 5 0.5444 1.5000 0.2990
edge 5 0.0444 -0.5000 0.7010
atom 6 4 0.95563 0.50000 0.29902
edge 6 1.0444 1.0000 0.2010
edge 6 1.0444 0.0000 0.2010
edge 6 1.0444 1.5000 0.7010
edge 6 0.5444 -0.5000 0.2990
atom 7 4 0.04437 0.00000 0.20098
edge 7 -0.0444 0.5000 0.2990
edge 7 -0.0444 -0.5000 0.2990
edge 7 -0.0444 1.0000 -0.2010
```

edge 7 0.4556 -1.0000 0.2010
atom 8 4 0.54437 0.00000 0.79902
edge 8 0.4556 0.5000 0.7010
edge 8 0.4556 -0.5000 0.7010
edge 8 0.4556 1.0000 1.2010
edge 8 0.9556 -1.0000 0.7990
end

---

8 - novel topology, **igc2**
(as obtained after simiplification of the structure from Cmcm to P1 space group and adjancy matrix correction to obtain the $AB^2B'^2$ 3D network)
Coordination sequences

Zn1: 1 2 3 4 5 6 7 8 9 10
Num 4 12 30 70 130 212 315 430 545 682
Cum 5 17 47 117 247 459 774 1204 1749 2431

Zn2: 1 2 3 4 5 6 7 8 9 10
Num 4 12 30 70 130 212 315 430 545 682
Cum 5 17 47 117 247 459 774 1204 1749 2431

Zn3: 1 2 3 4 5 6 7 8 9 10
Num 4 12 30 70 130 212 315 430 545 682
Cum 5 17 47 117 247 459 774 1204 1749 2431

Zn4: 1 2 3 4 5 6 7 8 9 10
Num 4 12 30 70 130 212 315 430 545 682
Cum 5 17 47 117 247 459 774 1204 1749 2431

Zn5: 1 2 3 4 5 6 7 8 9 10
Num 4 12 30 70 130 212 315 430 545 682
Cum 5 17 47 117 247 459 774 1204 1749 2431

Zn6: 1 2 3 4 5 6 7 8 9 10
Num 4 12 30 70 130 212 315 430 545 682
Cum 5 17 47 117 247 459 774 1204 1749 2431

Zn7: 1 2 3 4 5 6 7 8 9 10
Num 4 12 30 70 130 212 315 430 545 682
Cum 5 17 47 117 247 459 774 1204 1749 2431

Zn8: 1 2 3 4 5 6 7 8 9 10
Num 4 12 30 70 130 212 315 430 545 682
Cum 5 17 47 117 247 459 774 1204 1749 2431

TD10=2431

Vertex symbols for selected sublattice

Zn1 Point symbol:{6^5.8}

Extended point symbol:[6.6.6.6.6(2).8(2)]

Zn2 Point symbol:{6^5.8}
Extended point symbol:[6.6.6.6.6(2).8(2)]

Zn3 Point symbol:{6^5.8}
Extended point symbol:[6.6.6.6.6(2).8(2)]

Zn4 Point symbol:{6^5.8}
Extended point symbol:[6.6.6.6.6(2).8(2)]

Zn5 Point symbol:{6^5.8}
Extended point symbol:[6.6.6.6.6(2).8(2)]

Zn6 Point symbol:{6^5.8}
Extended point symbol:[6.6.6.6.6(2).8(2)]

Zn7 Point symbol:{6^5.8}
Extended point symbol:[6.6.6.6.6(2).8(2)]

Zn8 Point symbol:{6^5.8}
Extended point symbol:[6.6.6.6.6(2).8(2)]

Point symbol for net: {6^5.8}
4-c net; uninodal net

crystal
name C19 H20 N2 O4 Zn
cell 22.1641 9.8266 22.0654 90.000 90.000 90.000
group P1
atom 1 4 0.45563 0.00000 0.20098
edge 1 0.5444 -0.5000 0.2990
edge 1 0.5444 0.5000 0.2990
edge 1 0.5444 -1.0000 -0.2010
edge 1 0.0444 1.0000 0.2010
atom 2 4 0.95563 0.00000 0.79902
edge 2 1.0444 -0.5000 0.7010
edge 2 1.0444 0.5000 0.7010
edge 2 1.0444 -1.0000 1.2010
edge 2 0.5444 1.0000 0.7990
atom 3 4 0.04437 0.50000 0.70098
edge 3 -0.0444 0.0000 0.7990
edge 3 -0.0444 1.0000 0.7990
edge 3 -0.0444 -0.5000 0.2990
edge 3 0.4556 1.5000 0.7010
atom 4 4 0.54437 0.50000 0.29902
edge 4 0.4556 0.0000 0.2010
edge 4 0.4556 1.0000 0.2010
edge 4 0.4556 -0.5000 0.7010
edge 4 0.9556 1.5000 0.2990

atom 5 4 0.45563 0.50000 0.70098
edge 5 0.5444 1.0000 0.7990
edge 5 0.5444 0.0000 0.7990
edge 5 0.5444 1.5000 0.2990
edge 5 0.0444 -0.5000 0.7010
atom 6 4 0.95563 0.50000 0.29902
edge 6 1.0444 1.0000 0.2010
edge 6 1.0444 0.0000 0.2010
edge 6 1.0444 1.5000 0.7010
edge 6 0.5444 -0.5000 0.2990
atom 7 4 0.04437 0.00000 0.20098
edge 7 -0.0444 0.5000 0.2990
edge 7 -0.0444 -0.5000 0.2990
edge 7 -0.0444 1.0000 -0.2010
edge 7 0.4556 -1.0000 0.2010
atom 8 4 0.54437 0.00000 0.79902
edge 8 0.4556 0.5000 0.7010
edge 8 0.4556 -0.5000 0.7010
edge 8 0.4556 1.0000 1.2010
edge 8 0.9556 -1.0000 0.7990
end

8a - as obtained after UV irradiation of **8** for 6 hours - novel topology, **igc2**
Coordination sequences

Zn1: 1 2 3 4 5 6 7 8 9 10
Num 4 12 30 70 130 212 315 430 545 682
Cum 5 17 47 117 247 459 774 1204 1749 2431

TD10=2431

Vertex symbols for selected sublattice

Zn1 Point symbol:{6^5.8}
Extended point symbol:[6.6.6.6.6(2).8(2)]

Point symbol for net: {6^5.8}
4-c net; uninodal net

crystal
name ort_a.res in Pbcn
cell 9.8454 21.9051 21.9051 90.000 90.000 90.000
group Pbcn
atom 1 4 0.49776 0.29674 0.45427
edge 1 0.9978 0.2033 0.5457
edge 1 -0.0022 0.2033 0.5457
edge 1 1.5022 0.2967 0.0457
edge 1 -0.4978 0.7033 0.5457
end

---

9 - novel topology, **igc1**
Coordination sequences

Zn1: 1 2 3 4 5 6 7 8 9 10
Num 4 12 30 70 128 216 332 466 598 742
Cum 5 17 47 117 245 461 793 1259 1857 2599

Zn2: 1 2 3 4 5 6 7 8 9 10
Num 4 12 30 70 130 220 334 466 606 747
Cum 5 17 47 117 247 467 801 1267 1873 2620

Zn3: 1 2 3 4 5 6 7 8 9 10
Num 4 12 30 70 126 212 334 466 608 748
Cum 5 17 47 117 243 455 789 1255 1863 2611

TD10=2612

Vertex symbols for selected sublattice

Zn1 Point symbol:{6^5.8}
Extended point symbol:[6.6.6.6.6(2).8(2)]

Zn2 Point symbol:{6^5.8}
Extended point symbol:[6.6.6.6.6(2).8(2)]

Zn3 Point symbol:{6^5.8}
Extended point symbol:[6.6.6.6.6(2).8(2)]

Point symbol for net: {6^5.8}
4,4,4-c net with stoichiometry (4-c)(4-c)2(4-c); 3-nodal net

crystal
name C19 H22 N2 O4 Zn, 0.38(H2 O)
cell 24.6620 37.1800 8.7826 90.000 90.000 90.000
group C2221
atom 1 4 0.43029 0.50000 0.50000
edge 1 0.5697 0.5000 1.0000
edge 1 0.5697 0.5000 0.0000
edge 1 0.2403 0.2914 -0.5961
edge 1 0.2403 0.7086 1.5961
atom 2 4 0.24034 0.29141 0.40389
edge 2 0.2597 0.2086 -0.0961
edge 2 0.2597 0.2086 0.9039
edge 2 0.0000 0.4566 -0.7500
edge 2 0.4303 0.5000 1.5000
atom 3 4 0.00000 0.45663 0.25000
edge 3 0.0000 0.5434 0.7500
edge 3 0.0000 0.5434 -0.2500
edge 3 -0.2403 0.2914 -0.9039
edge 3 0.2403 0.2914 1.4039

end

References

S1. T. G. Mitina and V. A. Blatov, *Cryst. Growth Des.*, 2013, **13**, 1655.

S2. E. V. Alexandrov, A. P. Shevchenko, A. A. Asiri and V. A. Blatov, *CrystEngComm*, 2015, **17**, 2913.

S3. A. P. Shevchenko, I. A. Blatov, E. V. Kitaeva and V. A. Blatov, *Cryst. Growth Des.*, 2017, **17**, 774.

S4. Winn, M. D., Ballard, C. C., Cowtan, K. D., Dodson, E. J., Emsley, P., Evans, P. R., Keegan, R. M., Krissinel, E. B., Leslie, A. G. W., McCoy, A., McNicholas, S. J., Murshudov, G. N., Pannu, N. S., Potterton, E. A., Powell, H. R., Read, R. J., Vagin, A., Wilson, K. S., *Acta. Cryst.*, 2011, **D67**, 235.

S5. Sheldrick, G. M., *Acta Crystalogr.*, 2015, **C71**, 3.

S6. Dolomanov, O. V., Bourhis, L. J., Gildea, R. J., Howard, J. A. K., Puschman, H., *J. Appl. Cryst.*, 2009, **42**, 339.

S7. *Bruker TOPAS 5 User Manual*, Bruker AXS GmbH, Karlsruhe, Germany, 2014.

S8. R. W. Cheary, A. Coelho, *J. Appl. Crystallogr.* 1992, **25**, 109.

S9. M. Järvinen, *J. Appl. Crystallogr.* 1993, **26**, 525.
